# Sexual dimorphism in the mouse bone marrow niche regulates hematopoietic engraftment via sex-specific Kdm5c/Cxcl12 signaling

**DOI:** 10.1172/JCI182125

**Published:** 2025-01-21

**Authors:** Xiaojing Cui, Liming Hou, Bowen Yan, Jinpeng Liu, Cuiping Zhang, Pinpin Sui, Sheng Tong, Larry Luchsinger, Avital Mendelson, Daohong Zhou, Feng-chun Yang, Hui Zhong, Ying Liang

**Affiliations:** 1 Lindsley F. Kimball Research Institute, New York Blood Center, New York, New York, USA.; 2 Department of Toxicology and Cancer Biology, University of Kentucky, Lexington, Kentucky, USA.; 3 Department of Pharmacodynamics, College of Pharmacy, University of Florida, Gainesville, Florida, USA.; 4 Department of Internal Medicine, University of Kentucky, Lexington, Kentucky, USA.; 5 Department of Cell Systems & Anatomy, University of Texas Health at San Antonio, San Antonio, Texas, USA.; 6 Department of Bioengineering, University of Kentucky, Lexington, Kentucky, USA.; 7 Department of Biochemistry & Structural Biology, University of Texas Health at San Antonio, San Antonio, Texas, USA.

**Keywords:** Stem cells, Transplantation, Stem cell transplantation

## Abstract

The bone marrow (BM) niche is critical in regulating hematopoiesis, and sexual dimorphism and its underlying mechanism in the BM niche and its impact on hematopoiesis are not well understood. We show that male mice exhibited a higher abundance of leptin-receptor–expressing mesenchymal stromal cells (LepR-MSCs) compared with female mice. Sex-mismatched coculture and BM transplantation showed that the male BM niche provided superior support for in vitro colony formation and in vivo hematopoietic engraftment. The cotransplantation of male stromal cells significantly enhanced engraftment in female recipients. Single-cell RNA-seq revealed that the lower expression of the X-linked lysine H3K4 demethylase, *Kdm5c*, in male MSCs led to the increased expression of *Cxcl12*. In MSC-specific *Kdm5c*-KO mouse model, the reduction of KDM5C in female MSCs enhanced MSC quantity and function, ultimately improving engraftment to the male level. Kdm5c thus plays a role in driving sexual dimorphism in the BM niche and hematopoietic regeneration. Our study unveils a sex-dependent mechanism governing the BM niche regulation and its impact on hematopoietic engraftment. The finding offers potential implications for enhancing BM transplantation efficacy in clinical settings by harnessing the resource of male MSCs or targeting Kdm5c.

## Abstract 2

Sex dimorphism exists in the mouse bone marrow niche, and male stromal cells promote better hematopoietic engraftment in transplantation. Kdm5c-Cxcl12 signaling pathway contribute to the sex dimorphism in the niche and HSCT.

## Graphical abstract

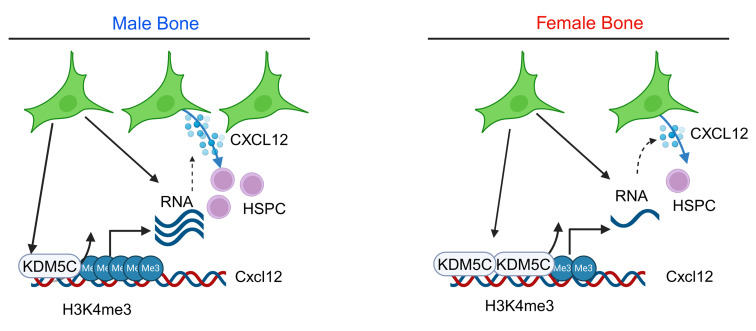

## Introduction

Hematopoietic stem cell transplantation (HSCT) or bone marrow (BM) transplantation is a life-saving and life-improving treatment for patients with blood diseases and immune disorders (1, 2). Allogenic and autologous HSCTs involve the transfer of hematopoietic stem cells (HSCs) into the recipient, with HSC homing and engrafting in the BM niche to regenerate the blood and immune system (3–5). HSCs, BM stromal cells, and their interaction within the niche are integral to the therapeutic efficacy (6). Current efforts to increase treatment effectiveness focus on risk variables associated with both donor and recipients, such as HLA matching, age, disease type, and stage. Gender mismatch is another crucial factor to consider, especially with allogeneic HSCT (7–12). Male donors to female recipients have been associated with a higher risk and severity of graft-versus-host disease (GVHD) (8), although another study suggests that male-to-female transplants may have lower relapse rates (10). Furthermore, studies have shown that HSCT from female donors to male recipients was associated with a lower relapse rate in patients with hematologic malignancies compared with all other donor-recipient gender combinations (11, 13–15). However, Kim et al. found that female donor cells, especially in female-to-male transplants, are associated with worse outcomes in overall survival and progression-free survival compared with male donor cells (7). These conflicting reports highlight the necessity for understanding the fundamental sex differences in hematopoiesis and BM stromal cells, which will thus be informative and instructive in clinical HSCT (16).

Sex variations in hematopoiesis have been found in mice and humans, which change over the course of their lives (17–20). Male mice have more BM hematopoietic stem and progenitor cells (HSPC) than female mice (21). Female HSCs divide more frequently and exhibit greater self renewal in response to estrogen (18). The aging process brings distinct changes, with females experiencing an expansion of HSPCs from a young age to middle age, while males exhibit this expansion from middle to old age. Sex hormones, including estrogen and follicle-stimulating hormone (FSH), have been identified as contributors to sexual dimorphism in HSPCs and aging hematopoiesis (21–25). In humans, women are found to have lower levels of circulating HSPCs than men (26). Furthermore, sex disparities extend to the human immune system (27). For instance, females consistently maintain higher numbers of CD4^+^ T cells and CD4-to-CD8 ratios throughout their lives, indicating a potential influence of sex chromosome-linked genetic factors (27–30). On the other hand, several features of innate immunity, such as proinflammatory responses and natural killer (NK) cell function, undergo changes at puberty and gradually decline in later life, suggesting the influence of sex hormones on these processes (27).

Most studies on sexual dimorphism in hematopoiesis have focused on HSPCs and immune cells. Little is known about the sex differences in the BM niche and the underlying mechanisms. The BM niche is critical in regulating HSC self renewal, differentiation, and engraftment (31–33). Previous research employing various in vivo mouse models, as well as recent single-cell sequencing, has revealed a comprehensive picture of niche cellular composition and its functional implications for HSCs (6, 34–36). Mesenchymal stem/stromal cells (MSCs) are a prominent component that produces key niche factors such as CXCL12 and stem cell factors (SCFs), both of which are important regulators of HSC homing and engraftment (37–40). Leptin-receptor–positive (LepR^+^) MSCs are identified as the one of the major resources for the high expression of SCF and CXCL12 (41). In this study, we find that male mice have more LepR-MSCs than females. Male BM stromal cells support better in vitro colony formation and in vivo hematopoietic engraftment. Single-cell RNA-seq shows more MSCs in male stroma. Mechanistically, we found that an X chromosome–linked histone demethylase gene, *Kdm5c*, had reduced expression in male MSCs, increasing H3K4me3, which leads to higher expression of *Cxcl12* in male stromal. MSC-specific *Kdm5c* knockout in females increases engraftment to male levels. The findings reveal a sex-dependent BM niche regulation mechanism and suggest that male MSCs or *Kdm5c* inhibition could be used in the clinic to improve HSCT.

## Results

### Sexual dimorphism in the BM niche and hematopoiesis.

**Figure 1:**
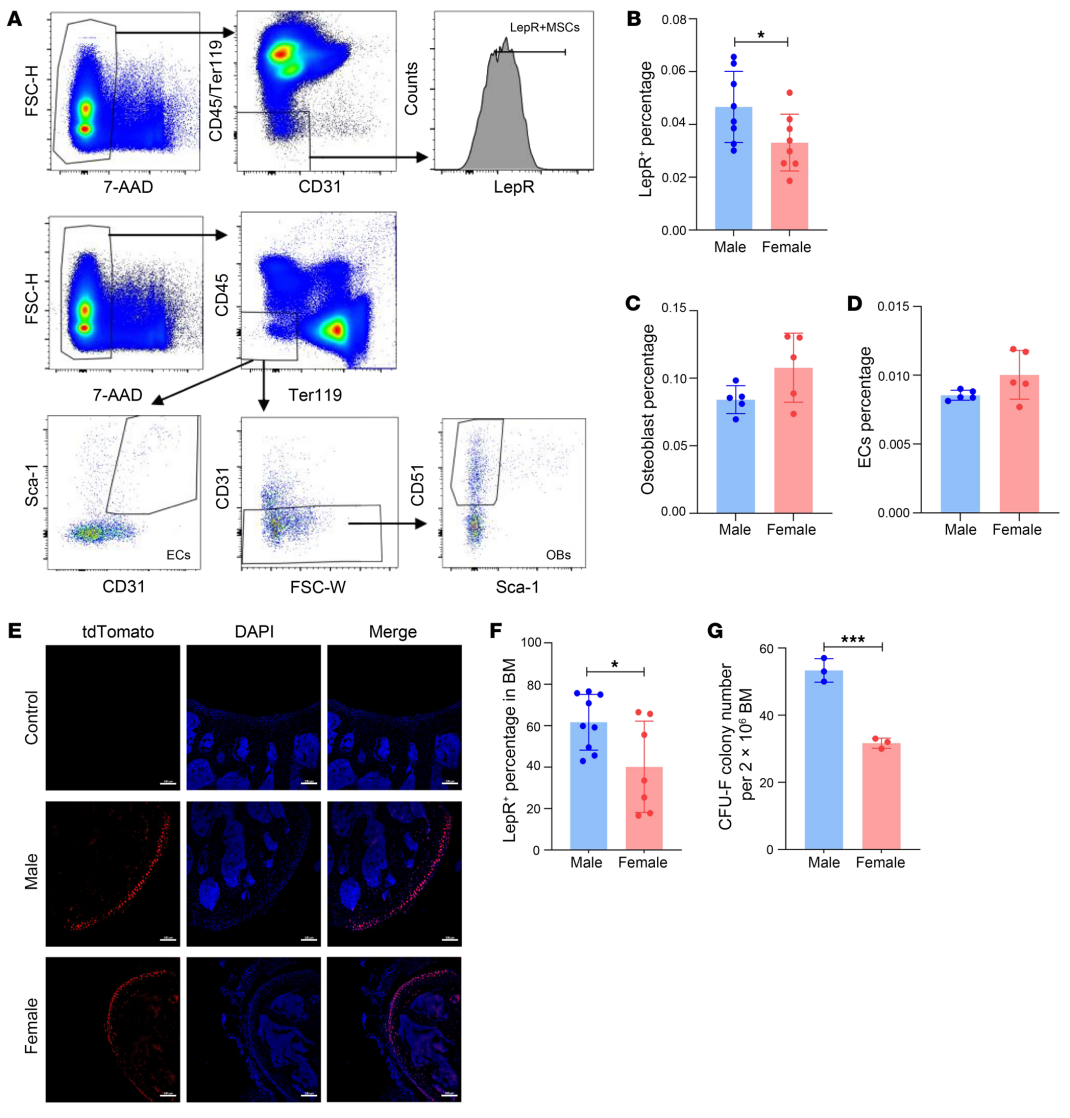
Sexual dimorphism in the BM niche. (**A**) Representative FACS analysis of stromal cells: MSCs are 7-AAD^–^, Ter119^–^, CD45^–^, CD31^–^, and LepR^+^; ECs are 7-AAD^–^, Ter119^–^, CD45^–^, CD31^+^, and Sca-1^+^; OBs are 7-AAD^–^, Ter119^–^, CD45^–^, CD31^–^, CD51^+^, and Sca-1^–^. (**B**–**D**) The percentage of (**B**) MSCs, (**C**) OBs, and (**D**) ECs in the male and female BM niches (*n* = 5–8). (**E**) Representative femur sections from WT, male and female LepR-tdTomato mice. Scale bar: 100 μm. (**F**) The percentage of LepR-tdTomato^+^ cells in male and female BM (*n* = 2, with at least 3 slices from each mouse). (**G**) The absolute number of clones, defined by the colony-forming units fibroblast (CFU-F) assay, was observed on day 14. Data presented are an average of 3 male or female mice for each group. All data were analyzed by a 2-tailed *t* test and shown as mean ± SD. *P < 0.05 ***P < 0.001.

To understand the sex difference in the BM niche, we used flow cytometry to identify the primary niche cells in male and female mice, including MSCs, endothelial cells (ECs), and osteoblasts (OBs) (Figure 1A). We used 2 types of markers to define MSCs: LepR^+^ (41) or PDGFRa^+^/Sca1^+^ (42) (Supplemental Figure 1A; supplemental material available online with this article; https://doi.org/10.1172/JCI182125DS1). We found that the majority of nonhematopoietic BM cells (CD45^–^ Ter119^–^ CD31^–^ cells are LepR^+^ (74.4%) whereas PDGFRa^+^/Sca1^+^ MSCs are only composed of 10.1%, which is consistent with previously report (42). Among LepR^+^ cells, 9.48% are PDGFRa^+^/Sca1^+^ MSCs, indicating that these 2 types of MSCs may maintain a large difference in their immunophenotype. Considering LepR^+^ MSC accounts for 94% of CFU-Fs in adult BM (41), LepR^+^ MSCs express high level of CXCL12 that is important to hematopoietic regulation (43), we therefore focused on LepR^+^ MSC in this study. The percentage of LepR^+^ MSCs in male mice was significantly higher than in female mice (Figure 1B), whereas ECs and OBs revealed no gender differences (Figure 1, C and D). By using LepR-tdTomato mice to quantify MSCs in situ (Figure 1E), we detected a similar increase in the number of MSCs (Figure 1F). The colony-forming fibroblast (CFU-F) assay further verified this finding (Figure 1G). We also compared the peripheral blood (PB) and BM hematopoiesis between male and female mice. Male mice had a lower percentage of T cells but a higher percentage of myeloid cells in PB (Supplemental Figure 1B). In the flow cytometric analysis of BM cells (Supplemental Figure 1C), male mice had more LSK (lineage^–^, Sca-1^+^, and c-kit^+^) cells and multipotent progenitor cells (MPPs), more long-term HSCs were quiescent, and fewer LSK cells underwent apoptosis than female in BM (Supplemental Figure 1, D–K), which is consistent with the previous reports (17, 18, 21). Overall, sexual differences exist in both stromal and hematopoietic cells.

### Male BM stroma provides superior support for in vitro colony formation and in vivo hematopoietic engraftment.

To examine the sex difference of MSCs in supporting HSPCs, we set up a gender mismatch long-term colony formation assay (Supplemental Figure 2A). Male or female nonhematopoietic BM cells (CD45^–^ Ter119^–^ CD31^–^) were cultured into confluency, the majority of which were Sca1^+^ MSCs with trilineage (adipo-, osteo- and chondro-) differentiation potential (Supplemental Figure 2, B and C). The confluent cells were then seeded into the 96-well plate for the colony formation assay (passage 2). Male or female HSPC-enriched Lin^–^/Sca 1^+^/ckit^+^ (LSK) cells were seeded onto the stromal cells. The long-term colony (cobblestone area) forming cells (CAFC) were evaluated at day 35 of culture. The result showed that the number of CAFC on day 35 was significantly higher in male stroma than female, regardless of whether male or female LSK cells were seeded (Supplemental Figure 2D).

**Figure 2:**
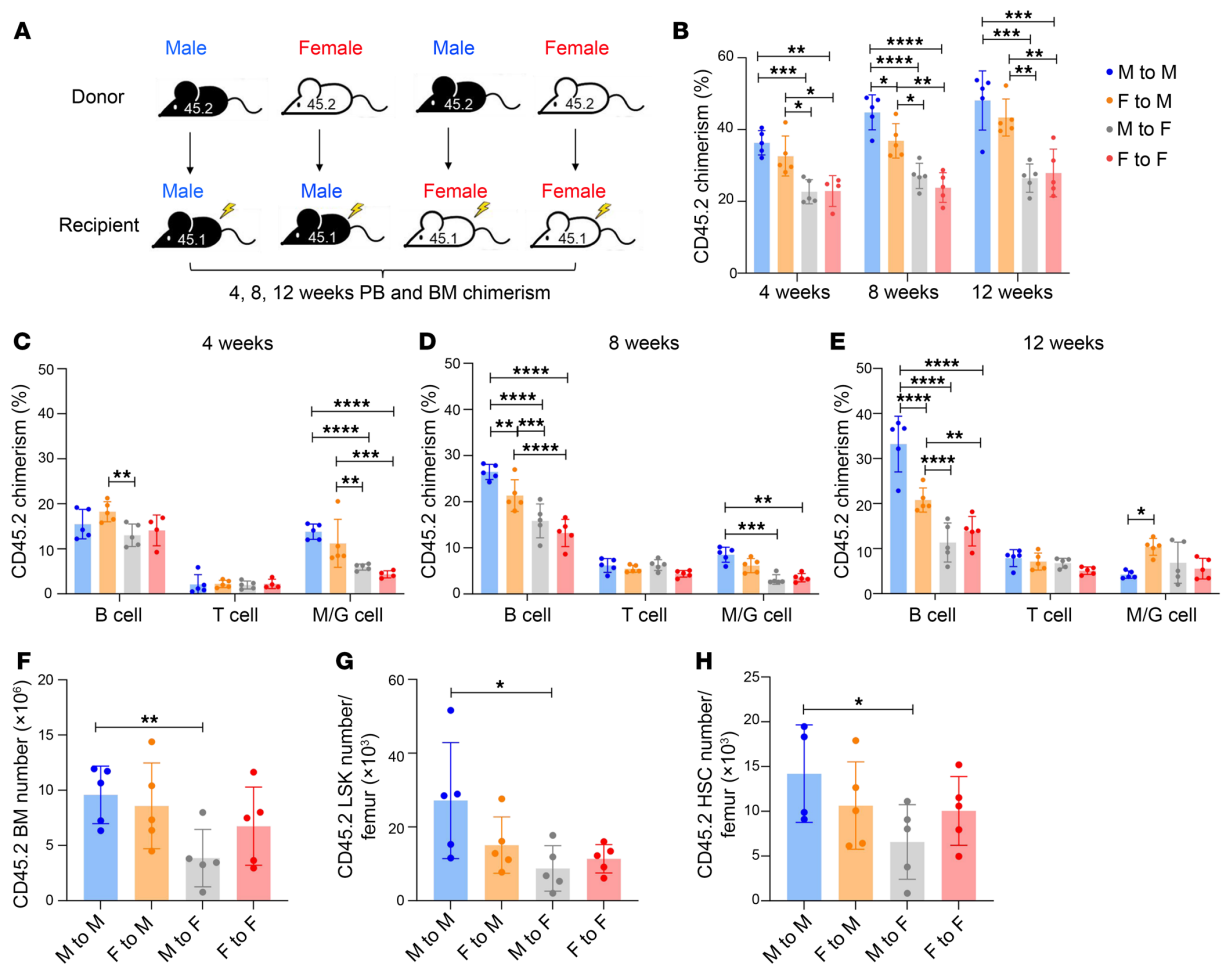
Male BM stroma provides better support for in vivo hematopoietic engraftment. (**A**) Experimental schematic for sex mismatch competitive transplantation assay. (**B**) Frequencies of male and female donor-derived (CD45.2) leukocytes from peripheral blood (PB) of male and female recipient mice (CD45.1) at 4, 8, and 12 weeks after transplantation. (**C**–**E**) Frequencies of male and female donor-derived B cells, T cells, and myeloid cells at (**C**) 4 weeks, (**D**) 8 weeks, and (**E**) 12 weeks after transplantation, obtained from the PB of male and recipient mice. (**F**–**H**) At 16 weeks after transplantation, the absolute number of the male and female donor-derived (**F**) BM, (**G**) LSK cells, and (**H**) HSC (LSK CD135^–^) cells in male and female recipient mice. The data were analyzed by 2-way ANOVA and shown as mean ± SD. *P < 0.05, **P < 0.01, ***P < 0.001, ****P < 0.0001.

We next performed sex mismatch BM competitive repopulation transplantation, in which 1 × 10^6^ donor BM cells from male or female BM (CD45.2) were mixed with an equal number of competitor BM cells of CD45.1 B6.SJL/BoyJ (the gender of the competitor is consistent with recipient) and retroorbitally injected into lethally (9Gy) irradiated male or female recipient mice (B6.SJL/BoyJ) (Figure 2A). Thus, 4 transplantation groups were set up (male to male, female to male, male to female and female to female). Percentages of donor cells (CD45.2) in PB cells were determined at 4, 8, and 12 weeks. Percentages of donor cells in BM were determined 12 weeks after transplantation. Blood chimerism in Figure 2B showed that male recipients had overall higher percentages of CD45.2-derived blood cells than female recipients at different time points after transplantation (blue and orange columns versus grey and red columns). In contrast, donor gender had either a milder (blue column versus orange column) or no effect (grey column versus red column) on blood repopulation. Moreover, male recipients had better myeloid engraftment at early (4 and 8 weeks) time points after transplant (Figure 2, C and D) but enhanced B cell engraftment later (8 and 12 weeks) (Figure 2, D and E) in comparison to female recipients. T lineage differentiation did not show any gender-specific difference. In BM, we found that male recipients showed overall better reconstitution in the populations of BM cells, LSK cells, and HSCs (long-term and short-term HSCs) than female recipients regardless of donor gender (Figure 2, F–H). These data suggest that male stroma promotes blood and BM HSPC engraftment, and stroma gender is more important than donor gender in determining engraftment efficiency.

### Male stroma promotes engraftment through a niche-extrinsic mechanism.

**Figure 3:**
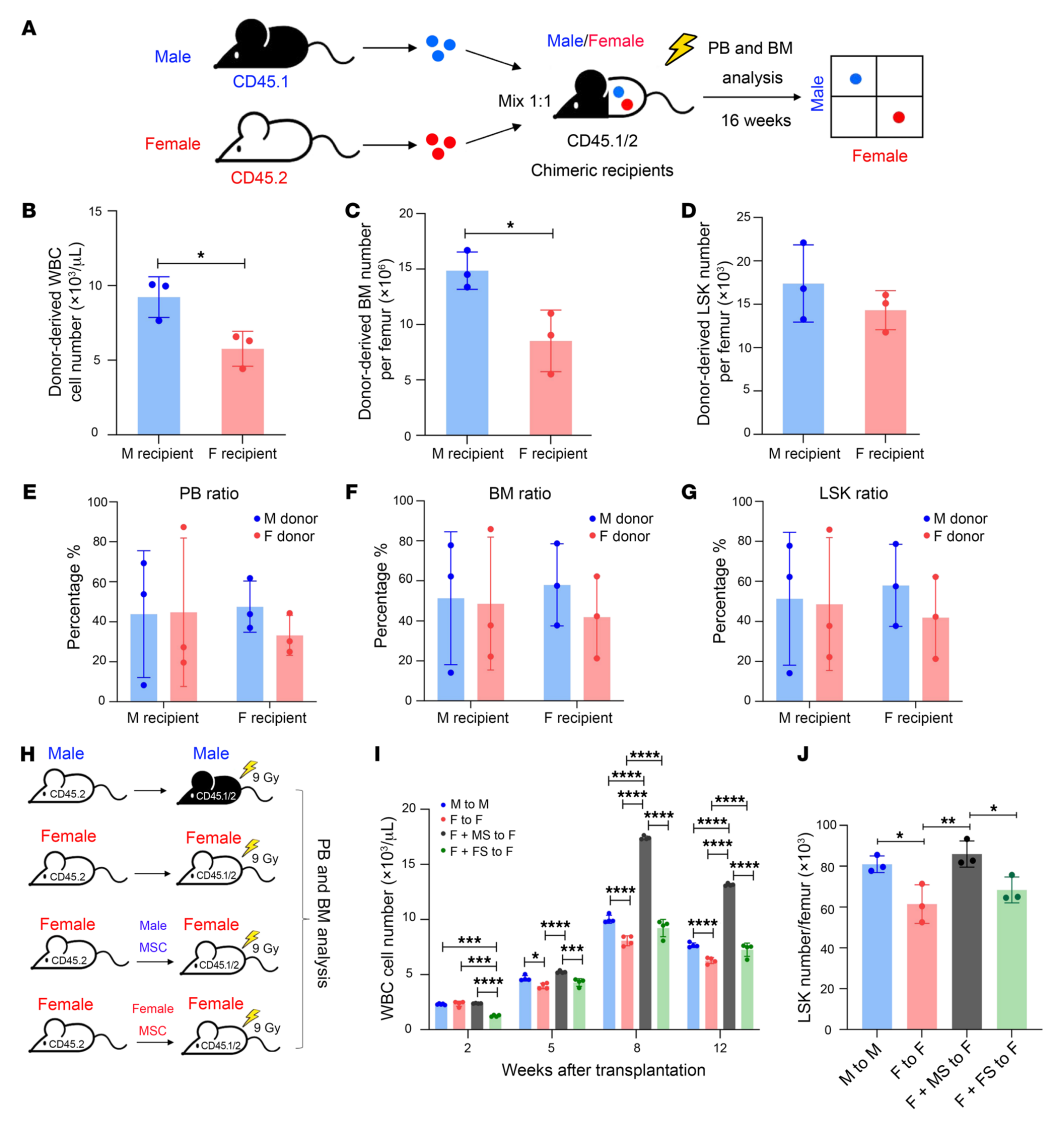
Male stroma promotes engraftment through a niche-extrinsic mechanism. (**A**) Experimental schematic for male and female donors’ mixture (1:1) were transplanted into CD45.1/2 chimeric mouse assay. (**B**) The absolute number of donor-derived leukocytes from PB of male and female recipient mice at 16 weeks after transplantation. (**C** and **D**) The absolute number of donor-derived (**C**) BM cells and (**D**) LSK cells in male and female recipient mice at 16 weeks after transplantation. The data were analyzed by a 2-tailed *t* test and were shown as mean ± SD. *P < 0.05. (**E**–**G**) The ratio between male and female donors of (**E**) WBC, (**F**) BM, and (**G**) LSK in male and female recipients. (**H**) Experimental schematic for BM cotransplanted with sorted mesenchymal stromal cells assay. (**I**) The absolute number of donor-derived leukocytes from PB of male and female recipient mice at 2, 5, 8, and 12 weeks after transplantation. (**J**) The absolute number of donor-derived LSK cells in male and female recipient mice at 16 weeks after transplantation. The data were analyzed by 2-way ANOVA, and shown as mean ± SD. *P < 0.05, **P < 0.01, ***P < 0.001, ****P < 0.0001.

To further dissect whether the sex difference in HSCT engraftment is caused by cell-intrinsic or niche-extrinsic mechanisms, we set a new transplantation model in which male and female BM cells competed in the same microenvironment. Male (CD45.1) and female (CD45.2) BM cells were mixed at a 1:1 ratio and transplanted into male or female CD45.1/CD45.2 chimeric mice, and blood and BM reconstitution were analyzed (Figure 3A). We found that male recipients had significantly higher donor-derived WBC counts, BM cellularity, and BM LSK cells than female recipients (Figure 3, B–D). Within the same gender recipient, the percentages of donor-derived cells in each population did not show differences in the gender chimerism (Figure 3, E–G), suggesting that male stroma promotes engraftment through a niche-extrinsic mechanism. To further confirm that male stromal cells can enhance engraftment, we cotransplanted male or female stromal cells (sorted 7-AAD^–^, CD45^–^, Ter119^–^, and CD31^–^ cells) with donor cells. 4 groups of transplantation were set up: (a) male donor to male recipient that showed the better engraftment; (b) female donor to female recipient that showed an overall lower engraftment; (c) female donor plus male stromal cells to female recipients; and (d) female donor plus female stromal cells to female recipients (Figure 3H). The result showed that male stromal significantly improved the recovery of white blood cell (WBC) counts (Figure 3I) and BM LSK cell number (Figure 3J). We further tested whether the transplanted MSC can successfully home to recipients’ BM. We isolated male or female MSCs (7-AAD^–^, CD45^–^, Ter119^–^ CD31^–^) from the BM of GFP^+^ mice and injected them to the recipient mice. After 1 week of transplantation, we checked the GFP signal in recipients’ BM and found GFP^+^ cells in both the M-MSC and F-MSC transplantation groups, suggesting that MSCs were successfully engrafted in the BM (Supplemental Figure 3A). Altogether, these data suggest that male stroma plays an important role in enhancing the engraftment efficiency in HSCT.

### The sex-specific differential expression of Cxcl12 underlies the sexual dimorphism in the BM niche.

**Figure 4:**
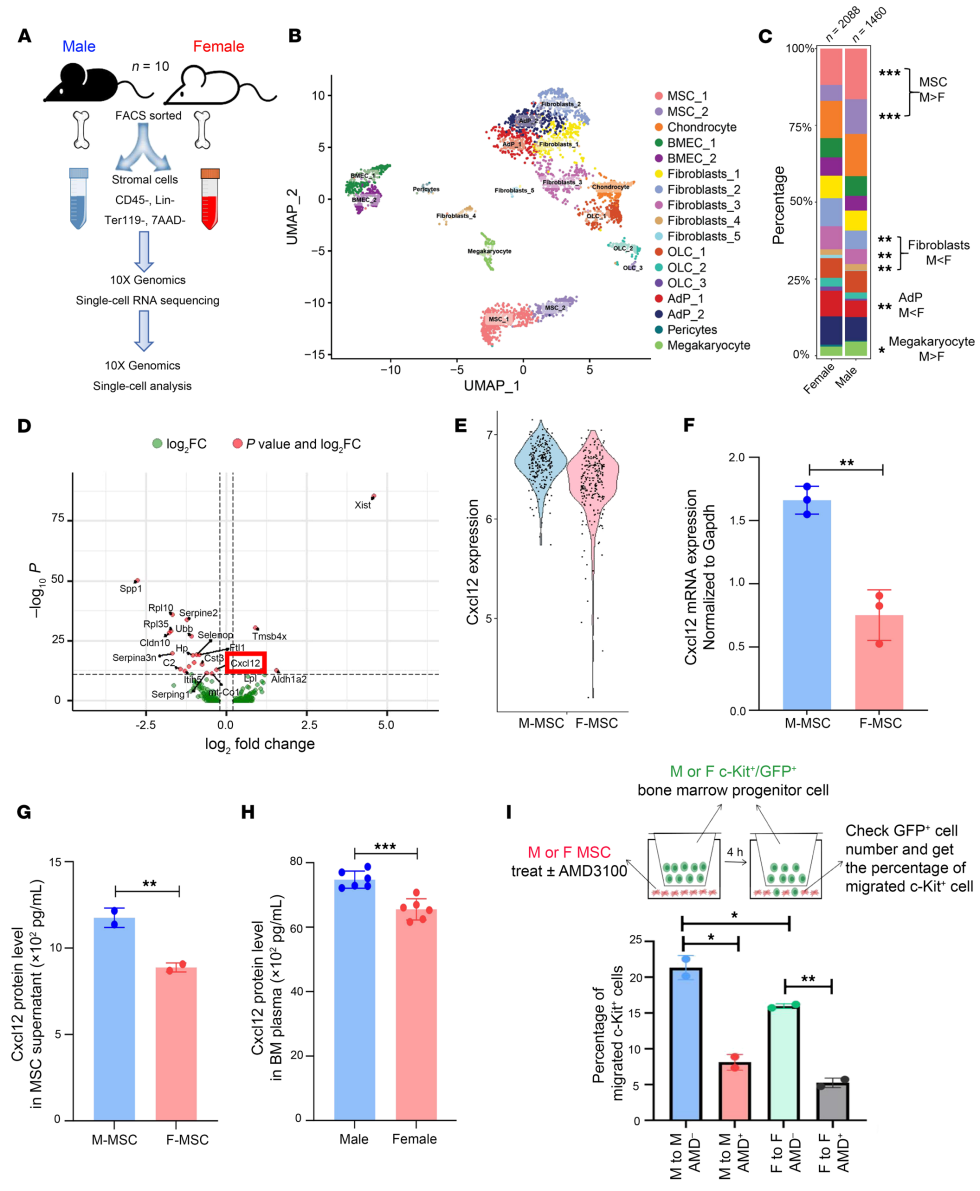
Differential expression of *Cxcl12* in male and female stromal cells. (**A**) Schematic illustration of the scRNA-seq analysis of male and female stromal cells. (**B**) UMAP plot of all stromal cells that passed the quality control test; 17 clusters were identified. (**C**) The percentage of each cluster in male and female stromal cells. (**D**) Volcano plot showing the specific DEGs (differentially expressed genes) in male and female MSC clusters. (**E**) The expression level of *Cxcl12* in male and female MSC clusters from scRNA-seq. (**F**) The mRNA level of *Cxcl12* in male and female MSC cells measured by real-time PCR. (**G**) The concentration of CXCL12 in male and female MSC culture supernatant detected by ELISA. (**H**) The concentration of CXCL12 in male and female BM plasma detected by ELISA. The data are shown as mean ± SD and analyzed by a 2-tailed t-test. (**I**) The migration rate of cKit^+^ cells was assessed using an in vitro transwell assay. The male or female MSC, treated with or without AMD3100 for 1 hour, were cocultured 4 hours with male or female GFP^+^/cKit^+^ cells, respectively. The migration rate was determined by the ratio of the number of migrated GFP^+^/cKit^+^ cells to the total number of cells. The data are shown as mean ± SD and analyzed by a 2 way ANOVA. *P < 0.05, **P < 0.01, ***P < 0.001, ****P < 0.0001.

To investigate the mechanism underlying the sexual dimorphism of BM niche and engraftment, we performed single-cell RNA–seq (scRNA-seq) of female and male BM stromal cells (Lin^–^, CD45^–^, Ter119^–^, and 7-AAD^–^) (Figure 4A) and identified 17 cell populations (Figure 4B). Male stroma had a much larger MSC population (high expression of LepR and Cxcl12) than female stroma (Figure 4C and Supplemental Figure 4A, top and middle panels), which is consistent with the result of immunophenotypically (LepR^+^) and functionally (CFU-F) characterized MSCs (Figure 1, B, F, and G respectively). In addition, we noticed that LepR^+^ MSCs were different from traditionally defined PDGFRa^+^/Sca1^+^(Ly6a) MSCs in which LepR^+^ MSCs express high level of Cxcl12, whereas PDGFRa^+^/Sca1^+^(Ly6a) are more like fibroblastic MSCs (Supplemental Figure 4B and Figure 4B) although both can produce CFU-F. These findings are consistent to the literature reports (34, 41–43). However, both types of cells can produce CFU-F, suggesting that they may function similarly, although immunophenotypes are different. Moreover, males had fewer fibroblasts (high *S100a4* expression) and adipocytes (high *Adipoq* expression) but more megakaryocytes (high *Plek* and *Pf4* expression) (Figure 4C). A total of 370 genes were differentially expressed between female and male stromal cells, and each stromal population had their unique gene signature (Supplemental Tables 1 and 2). We focused on the genes that were differentially expressed in MSCs because they are the most abundant stromal component, and it is the mostly significantly enriched in males than females. Among 142 differentially expressed genes between male and female MSCs, we identified *Cxcl12* (*Sdf-1*) and *Spp1* (osteopontin, *Opn*) as the top candidates (Figure 4D). *Cxcl12* and *Spp1* are important niche factors that mediate HSC-niche interaction and regulate HSC migration (homing and mobilization). Male MSCs had a higher expression of *Cxcl12* than female MSCs, according to scRNA sequencing and real-time PCR (Figure 4, E and F). Its protein level was higher in male MSC culture supernatant (Figure 4G) and BM fluid (Figure 4H) detected by ELISA. However, no difference in SPP1 expression was identified between male and female BM fluid (Supplemental Figure 4C). As a result, we thus focused on the *Cxcl12*. CXCL12 is important in HSC migration (33, 38–40, 44–50). To further confirm that CXCL12 contributes to the sexual dimorphism of the BM niche, we set up an in vitro HSPC migration assay. We isolated c-Kit^+^ cells from both male or female GFP mice, seeded them on the top of a transwell (5 μm), and cocultured with male or female MSCs pretreated with or without 5 μM AMD3100 (antagonist of CXCL12’s receptor CXCR4) for 1 hour. We next measured the percentages of GFP^+^ cells present at the bottom of transwell at 4 hours after coincubation. We found that the percentage of migrating c-Kit^+^ cells was significantly higher in male stromal cells than female ones (blue versus green column). Blocking the function of CXCL12 with AMD3100 reduced the percentage of migrating c-Kit^+^ /GFP^+^ cells in both genders and eliminated their difference (Figure 4I). These data suggest that male stromal cells can promote better hematopoietic engraftment via CXCL12-mediated increase in migration.

### The sex chromosome gene Kdm5c regulates the sex-specific differential expression of Cxcl12 in the BM niche.

**Figure 5:**
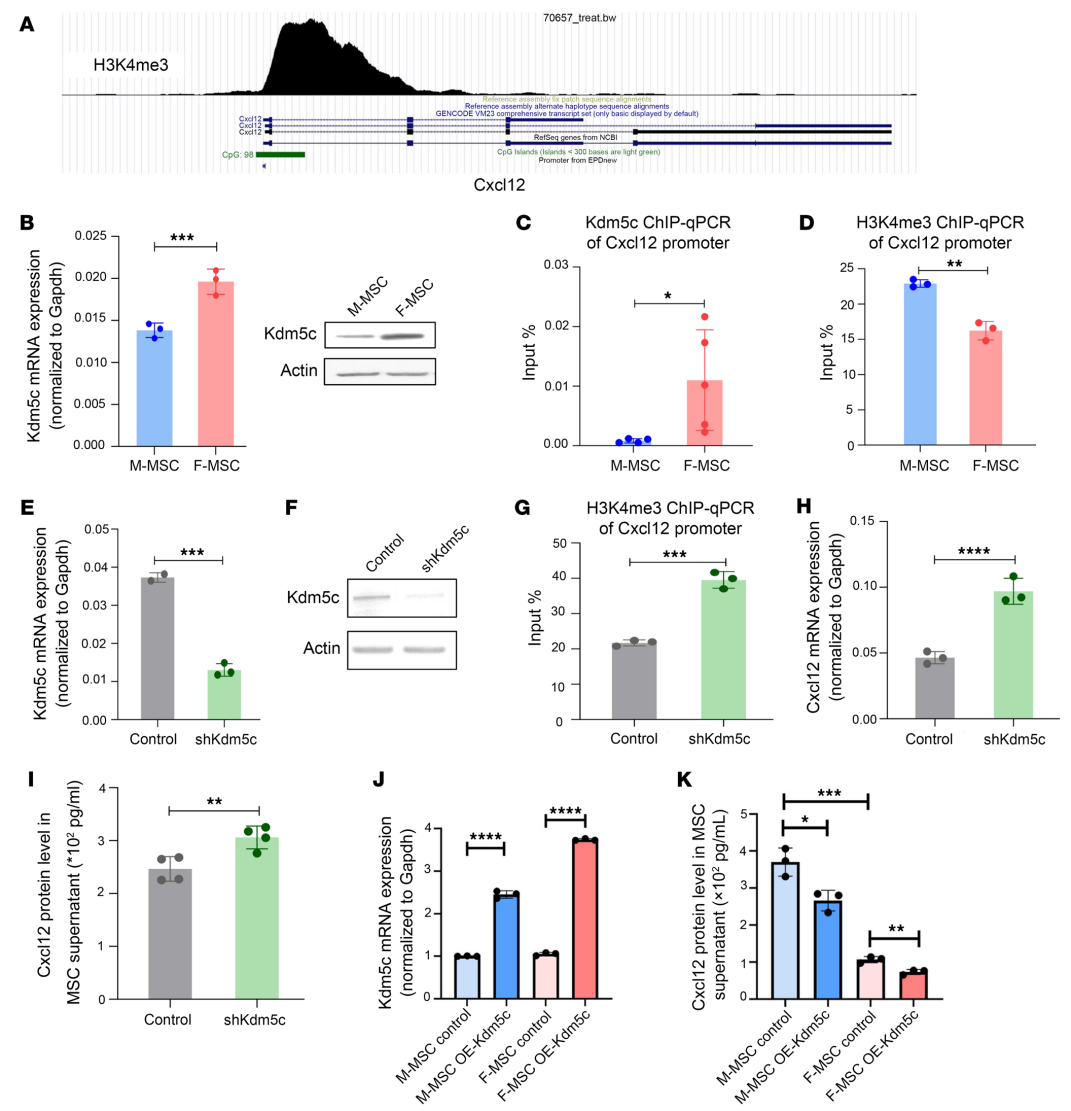
The sex chromosome gene *Kdm5c* contributes to the sex-specific differential expression of *Cxcl12* in the BM niche. (**A**) H3K4me3 binding to the CpG enrichment region of *Cxcl12* from the ChIP-seq data of UCSC and Cistrome Database. (**B**) The mRNA and protein level of KDM5C in male and female MSC cells. (**C**) ChIP-qPCR on male and female MSC cells for *Cxcl12* promoter region using the antibody against KDM5C. (**D**) ChIP-qPCR on male and female MSC cells for *Cxcl12* promoter region using the antibody against H3K4me3. (**E**) The mRNA level of *Kdm5c* in female MSC cells with control or *shKdm5c* transduction. (**F**) The protein level of KDM5C in female MSC cells with control or *shKdm5c* transduction. (**G**) ChIP-qPCR on female MSC cells treated with control or *shKdm5c* for *Cxcl12* promoter region using the antibody against H3K4me3. (**H**) *Cxcl12* mRNA level in control or *shKdm5c* transduced female MSC detected by real-time PCR. (**I**) The protein level of CXCL12 in female MSC cells with control or *shKdm5c* transduction detected by ELISA. The data were derived from 2 independent experiments with 2–3 replicates from each experiment, and were shown as mean ± SD, and analyzed by 2-tailed *t* test. (**J**) The mRNA level of *Kdm5c* in both male and female MSC with control or overexpressing (OE) *Kdm5c* plasmid. (**K**) The concentration of CXCL12 in male and female MSC culture supernatant overexpressed (OE) with or without *Kdm5c* was detected by ELISA. The data were derived from 2 independent experiments with 2–3 replicates from each experiment, were shown as mean ± SD, and were analyzed by 2 way ANOVA. *P < 0.05, **P < 0.01, ***P < 0.001, ****P < 0.0001.

To determine the mechanisms underlying the sex-specific differential expression of *Cxcl12* in BM MSCs, we analyzed the *Cxcl12* promoter (http://cistrome.org/db/#/ and https://genome.ucsc.edu) and found a H3K4me3-enriched region that may be involved in *Cxcl12* transcriptional regulation (Figure 5A). We next asked which sex-specific genes could influence *Cxcl12* expression. KDM5C attracted our attention for the following reasons: (a) it is a histone demethylase specific for histone 3 lysine 4 di- and tri-methylation (H3K4me2/3) (51); (b) it is located on the X chromosome and escaped X chromosome inactivation (52, 53), implying that female cells should have nearly twice the expression of male cells; and (c) a significant portion of differentially expressed genes between male and female MSCs overlapped with genes whose expression was altered by KDM5C overexpression (54) (Supplemental Figure 4D). We hypothesize that KDM5C regulates sex-specific differential expression in *Cxcl12*. We first confirmed that *Kdm5c* expression was higher in female MSCs than in male MSCs at the mRNA and protein levels (Figure 5B), even though scRNA-seq revealed no difference (Supplemental Figure 4A, bottom panels). It could be due to a low Kdm5c expression in MSCs. ChIP-qPCR with KDM5C antibody showed its higher abundance in the *Cxcl12* promoter in female MSCs than in male MSCs (Figure 5C). In contrast, less H3K4me3 binds to the *Cxcl12* promoter in female MSCs than male MSCs (Figure 5D), suggesting the negative association between KDM5C and H3K4me3 in the *Cxcl12* promoter. To further validate that KDM5C regulates *Cxcl12* gene expression via modulating H3K4me3, we knocked down *Kdm5c* in female MSCs (Figure 5, E and F) and found that KDM5C knockdown enhanced H3K4me3 binding to the *Cxcl12* promoter (Figure 5G), resulting in the increased *Cxcl12* transcription and protein expression (Figure 5, H and I). We performed the same experiments in male MSCs and found a similar regulation of KDM5C in *Cxcl12* expression (Supplemental Figure 5, A–D). Furthermore, we overexpressed *Kdm5c* on both male and female MSCs (Figure 5J) and found that overexpressing of *Kdm5c* decreased the secreted CXCL12 protein level (Figure 5K). *Kdm5d* is the homolog of *Kdm5c* on the Y chromosome (52, 55). We asked whether it is also involved in the *Cxcl12* transcriptional regulation. The *Cxcl12* expression was unaffected by *Kdm5d* knockdown in male MSCs (Supplemental Figure 5, E–H). Altogether, these results imply that KDM5C regulates the transcription of *Cxcl12* via H3K4me3, which accounts for sex-specific differential expression of Cxcl12.

### Kdm5c contributes to the sexual dimorphism in MSC function and hematopoietic engraftment.

**Figure 6:**
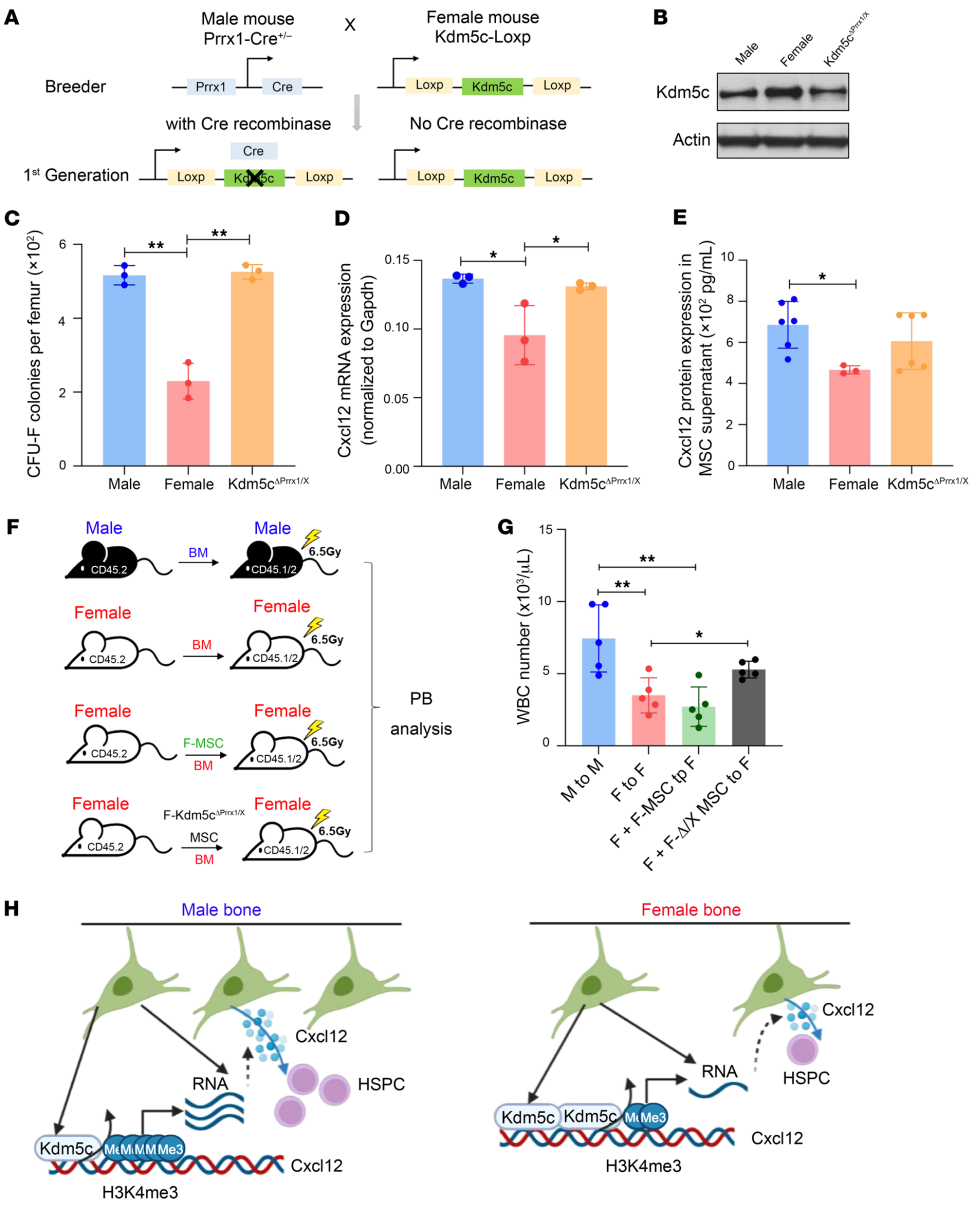
KDM5C contributes to the sexual dimorphism in MSC function and hematopoietic engraftment. (**A**) Schematic illustration shows the breeding strategy to obtain *Kdm5c*^ΔPrrx1/X^ mice. (**B**) The protein level of KDM5C in male (*Kdm5c*^loxp/Y^), female (*Kdm5c*^loxp/X^), and female *Kdm5c*^ΔPrrx1/X^ MSC cells. (**C**) The absolute number of clones in male (*Kdm5c*^loxp/Y^), female (*Kdm5c*^loxp/X^), and female *Kdm5c*^ΔPrrx1/X^ mice, defined by the colony-forming unit fibroblast (CFU-F) assay, was observed on day 14. (**D**) The mRNA level of *Cxcl12* in male, female, and female *Kdm5c*^ΔPrrx1/X^ MSC cells. (**E**) The concentration of CXCL12 in male, female, and female *Kdm5c*^ΔPrrx1/X^ MSC cell supernatants detected by ELISA. (**F**) Experimental schematic for the assay involving BM cotransplanted with *Kdm5c*^ΔPrrx1/X^ MSC cells. (**G**) The absolute number of leukocytes from PB in recipient mice at 8 weeks after transplantation. All the data are shown as mean ± SD and analyzed by 1-way ANOVA. (**H**) Male mice have more HSCs and MSCs than female mice. KDM5C was highly enriched in the *Cxcl12* promoter in female MSCs compared with male MSCs, leading to the increased level of demethylated H3K4me3, thus reducing the expression of *Cxcl12*. A lower level of KDM5C in male MSC leads to higher expression of *Cxcl12*, which promotes HSC engraftment and maintenance. *P < 0.05, **P < 0.01.

To investigate the role of *Kdm5c* in regulating sex-specific differences in MSC function and hematopoietic engraftment, we crossed the Prrx1-Cre line, which covers a broader range of stromal cells (38, 40, 56), with *Kdm5c*^fl/fl^ line to generate MSC-specific *Kdm5c* heterogenous conditional knockout female mice (*Prrx1-Cre; Kdm5c^fl/X^*, referred to as *Kdm5c*^ΔPrrx1/X^) (Figure 6A). We tested the hypothesis that depletion of a *Kdm5c* allele in female MSC would convert it to a male-like function. We confirmed that KDM5C was reduced by nearly half in female *Kdm5c^ΔPrrx1/X^* MSCs (Figure 6B). Female *Kdm5c*^ΔPrrx1/X^ had a higher number of CFU colonies than female control mice, reaching a level comparable to male control mice (Figure 6C). We further found that *Cxcl12* mRNA and protein expression were reverted to male levels in female *Kdm5c*^ΔPrrx1/X^ MSCs (Figure 6D and 6E). To further determine the effect of KDM5C reduction on engraftment, we sorted MSCs from female *Kdm5c*^ΔPrrx1/X^ mice, cotransplanted them with donor cells, and monitored the blood WBC recovery (Figure 6F). The result showed that female *Kdm5c*^ΔPrrx1/X^ MSCs significantly improved WBC recovery, similar to male-derived MSCs (Figure 6G). We also analyzed the hematopoiesis of *Kdm5c*^ΔPrrx1/X^ mice and found no significant changes in the blood cells and BM HSPC populations (Supplemental Figure 6). This indicates that *Kdm5c*^ΔPrrx1/X^ MSC may improve the homing of transplanted cells, which is consistent to the major role of CXCL12.

## Discussion

Sexual dimorphism in hematopoiesis has been discovered in HSPCs and immune cells in mice and humans during their lives (17–20). The BM niche plays important roles in the regulation of HSC function and hematopoiesis (6, 16, 37, 57). However, little is known about the sex differences in the BM niche and how they affect HSPC function and engraftment. In this study, we comprehensively characterize the differences between male and female BM niches and reveal several new findings. First, male BM has more MSCs than female BM, which were identified by immunophenotype (LepR^+^), colony formation (CFU-F) and scRNA sequencing (LepR^hi^Cxcl12^hi^). Secondly, male stroma provides the better support for hematopoietic engraftment and HSPC reconstitution than female stroma in HSCT. Thirdly, male MSCs have nearly half the dose of KDM5C expression compared with female cells, resulting in a higher level of CXCL12. Finally, knockout of 1 allele of *Kdm5c* in female stroma cells reverts its function to the male-like level, suggesting that KDM5C contributes to the sexual dimorphism in the BM niche and hematopoietic engraftment in HSTC.

*Kdm5c* is an X-linked gene encoding a histone demethylase specific to H3K4me2/3. It functions as a transcriptional repressor by removing active H3K4me2/3 marks from promoters (51, 58, 59). As an epigenetic and transcriptional regulator, KDM5C impacts various cellular processes in both males and females (54, 60, 61). Its mutations or dysregulation have been associated with certain intellectual disabilities, developmental delays, and various cancers (61–63). Physiologically, *Kdm5c* consistently escapes X inactivation in adult mouse cells and tissues, resulting in nearly double the dose of expression in female cells than in male cells (52, 64). However, whether such expressional disparity plays a role in the sexual dimorphism is not well known. Here, we showed that KDM5C contributes to sex-specific differences in the BM niche and its effect on hematopoietic engraftment. Deletion of 1 *Kdm5c* allele in female stroma increases the MSC number, *Cxcl12* expression, and blood cell recovery to the male level, implying the dose-dependent and sex-specific effect of KDM5C on BM MSCs. The sex-dependent regulation of KDM5C on osteoclastogenesis and BM mass was recently reported (65). Its higher expression was also shown to contribute to the increased adiposity in females (66). Osteoclasts and adipocytes are important BM niche cells (36, 67). Thus, our results, along with those of others, point to a broader role for KDM5C in determining the sex difference in different types of BM stromal cells. We further identified *Cxcl12* as the downstream signaling of KDM5C in MSCs. KDM5C regulates *Cxcl12* expression by modulating H3K4me3 activity. In the male niche, less KDM5C is enriched in the *Cxcl12* promoter, resulting in increased occupancy of H3K4me3 to the *Cxcl12* promoter, which enhances its expression. In the female niche, more KDM5C occupies the *Cxcl12* promoter, leading to less H3K4me3, which decreases its expression (Figure 6H). Thus, KDM5C, H3K4me3, and CXCL12 signaling pathway demonstrate the sex-specific and dose-dependent effect on sexual dimorphism in the BM niche.

CXCL12 is a chemoattractant that guides HSPCs to their specific niche and is involved in HSPC homing (50). In HSPCs, CXCL12 binds to CXCR4 to retain HSPCs within the niche and maintain self-renewal and quiescence (40, 46, 48, 49). Thus, high CXCL12 level in male recipients or male MSCs may contribute to the increased hematopoietic engraftment and HSPC reconstitution. Our study suggests that CXCL12-mediated homing could be 1 of the underlying mechanisms. However, we cannot exclude the possibility that other niche factors are also involved in the niche sex dimorphism because KDM5C has a broader impact on the epigenetic and transcriptional regulation. In addition, although the current study focuses on sex chromosome genes, we cannot exclude the possibility that sex hormones may also play a role in shaping the differences between male and female BM niches. However, the result of in vitro colony formation with sex-mismatched setting (Supplemental Figure 2, A and D) offer a way to avoid the influence of hormones, providing a valuable comparison to the in vivo transplantation scenarios. Future research into transplantation in ovariotomy or testiculectomy conditions will help to better understand the synergistic and/or independent role of sex chromosome genes and hormones.

Our study reveals differences in cellular composition and gene expression between male and female BM niches at the single-cell level. Male niche contains more MSCs and megakaryocytes (MKs), whereas female niche contains more fibroblasts and adipolineages (AdPs). MSCs and MKs are generally recognized for their supportive role in the regulation of normal hematopoiesis, whereas fibroblasts and AdPs play a more negative role (67, 68). This may explain why male mice have more HSPCs than female mice and regenerate hematopoiesis better as transplant recipients. Although our study is focused on the MSC and KDM5C-CXCL12 pathway, other signaling in specific stromal cell populations, such as ribosome biogenesis and extracellular matrix regulation (as shown in Supplemental Tables 1 and 2), could also involve in the sex dimorphism of BM niche. In addition, this study focuses on the gender differences in LepR+ MSCs and their functional effects on HSCT. It is possible that MSCs identified by other immunophenotypes, such as N-cadherin+ MSCs or PDGFRα+Sca1+ MSCs (43, 69), may also contribute to sex-based differences in the BM niche, warranting further investigation in future studies.

Gender mismatch in donor cells is an important factor to consider especially in allogenic HSCT. But it is difficult to conclude whether it is beneficial or not. While 1 study found a higher risk and severity of GVHD in female recipients who received male donors, another found a lower rate with relapse. MSCs have been shown to promote engraftment and reduce GVHD in clinical HSCT (70–72). However, not much is known about whether male or female derived MSCs affect the transplantation efficacy and outcome. Our findings show that male MSCs benefit HSCT in several ways, including: (a) improving white blood cell recovery and engraftment; (b) stimulating neutrophil and myeloid cell recovery early after HSCT and B lymphocyte recovery later; and (c) enhancing BM cell and HSPC reconstitution. Although transplantation in our mouse model is synergistic, our findings suggest that incorporating male derived MSCs into donor cells may further enhance the HSCT treatment efficacy. Moreover, pharmaceutical inhibition of KDM5C in either female stromal cells or even female recipients may be another way to enhance the HSCT efficacy. Our results also indicate that male donor to male recipient HSCT may be a better sex match, which could be informative to clinics when gender mismatch is a concern. 1 limitation in this study is that we cannot rule out the possibility that part of our phenotype is due to the immunological rejection from different genders. Further studies are needed to explore the immune system/reaction’s difference between genders in the context of gender mismatched transplantation and hematological malignancy disease models. Overall, our study not only provides important insights into the cellular and molecular basis of BM niche sex dimorphism, but also offer some practical information for clinic HSCT.

## Methods

### Sex as a biological variable.

In all mouse studies, both male and female mice were used. Sex was considered as a biological variable in the statistical analyses.

### Mice.

C57BL/6 (CD45.2) mice (Strain #:000664), B6.SJL/BoyJ (CD45.1) mice (Strain #:002014), and B6. Cg-Tg (Prrx1-cre) 1Cjt/J mice (Strain #:005584) were purchased from The Jackson Laboratory. Kdm5c^fl/fl^ mice (73) were provided by Harvard University (Boston, Massachusetts, USA), where Yang Shi (University of Oxford, Oxford, United Kingdom) originally generated this mouse line. LepR-tdTomato and GFP^+^ mice were provided by Avital Mendelson (New York Blood Center). To obtain the CD45.1/2 chimeric mice, the CD45.1 mice were crossed with CD45.2 mice. To achieve the tissue-specific deletion of Kdm5c, the Kdm5c^fl/fl^ mice were crossed with Prrx1-Cre mice. All the strains were maintained on a C57BL/6 background. All mice used were age-matched male and female and were 8–16 weeks old. For radiation, mice were exposed to a lethal (9Gy) dose or sublethal (6.5Gy) dose of total body irradiation in a Mark 1 irradiator (137 Cesium) (J.L. Shepherd & Associates) at a rate of 1.0 Gy/min with attenuator, on a rotating platform.

### PB, HSPC, and stromal cell identification and analysis by flow cytometry.

For stem and progenitor cell analysis, BM cells were obtained from the femur by flushing the central cavity with 2% FBS HBSS. Single-cell suspensions were washed and stained with the antibody lineage cocktails (BD Biosciences, Biotin Rat Anti-Mouse CD5, Cat#: 553019; BD Biosciences, Biotin Rat Anti-Mouse CD45R/B220, Cat#: 553086; BD Biosciences, Biotin Rat Anti-CD11b, Cat#: 553309; BD Biosciences, Biotin Rat Anti-Mouse CD8a, Cat#: 553029; BD Biosciences, Biotin Rat Anti-Mouse Ly-6G and Ly-6C, Cat#: 553125; BD Biosciences, Biotin Rat Anti-Mouse TER-119/Erythroid Cells, Cat#: 553672), anti-Sca-1 (eBioscience, Cat#: 25-5981-82), anti-cKit (BD Biosciences, Cat#: 553356), anti-CD135 (BD Biosciences, Cat#: 553842), anti-FcγR (CD16/32) (BD Biosciences, Cat#: 560540), anti-CD127 (eBioscience, Cat#: 48-1271-82), anti-CD34 (BD Biosciences, Cat#: 553733), and Streptavidin (for lineage cocktails, BD Biosciences, Cat#: 554063). PB was collected from the retroorbital venous plexus by using an EDTA-treated capillary. A complete blood count was performed on Hemavet 950 (Drew Scientific). PB lineage chimerism staining was performed with the following antibodies: antibody anti-CD45.1 (BD Biosciences, Cat#: 558701), anti-CD45.2 (BD Biosciences, Cat#: 561874), anti-B220 (for B cells) (BD Biosciences, Cat#: 552094), anti-CD90.2 (for T cells) (eBioscience, Cat#: 25-0902-82), anti-Gr-1 (BD Biosciences, Cat#: 553128), and anti-CD11b (for myeloid cells) (eBioscience, Cat#: 45-0112-82) (74). For the cell apoptosis analysis, BM cells were stained with apoptotic marker, anti-Annexin V (BD Biosciences, Cat#: 556547), and 7-AAD (7-amino actinomycin D) (Biosciences, Cat#: 559925) (75). For cell cycle analysis, BM cells were isolated and stained with HSPC markers as described above, then fixed and permeabilized by using the Fix and Perm kit from BD Pharmingen, followed by staining with anti-Ki-67 (BD Biosciences, Cat#: 556003) and 7-AAD. For stromal cell identification, hematopoietic and nonhematopoietic cells, located in both the perivascular (marrow) and endosteal niches (digested bones), were obtained based on our previously established method (76). Single-cell suspensions were washed and stained with antibodies CD45 (BD Biosciences, Cat#: 559864), Ter 119 (BD Biosciences, Cat#: 557909 (APC) or Cat#: 557915 (FITC)), CD31 (Biolegend, Cat#: 102422), Lepr (R&D Systems, Cat#: BAF497), CD51 (BD Biosciences, Cat#: 551187), PDGFRα (Biolegend, Cat#: 135916), Sca-1 (eBioscience, Cat#: 25-5981-82), Streptavidin (for Lepr, BD Biosciences, Cat#: 554063), and 7-AAD (35). Flow cytometry was performed and analyzed on the BD LSR II and BD Symphony A3 Cytometers.

### CFU-F assay.

Hematopoietic and nonhematopoietic cells were obtained from BM as described above. A total of 2 × 10^6^ cells were plated in a 60 mm tissue culture dish in 8 mL α-MEM supplemented with 20% heat-inactivated FBS and 1% penicillin/streptomycin. Two days and 7 days after incubation, the nonadherent cells were removed by washing with warm PBS, then renewed with the fresh medium. On day 14, cells were fixed with methanol and then stained with Giemsa. The dishes were air-dried and the colonies were counted. The frequency of CFU-F is defined as the ratio of the number of colonies to the number of cells seeded.

### Bone section, staining, and confocal imaging.

LepR-tdTomato male and female mice were used for bone imaging. Freshly dissected bones were fixed in ice-cold 4% paraformaldehyde at 4°C overnight followed by 10 days decalcification in 10% EDTA (pH = 7.2–7.4, at 4°C, with EDTA changes every 2–3 days). The specimens were then washed (1 hour total, 3 washes in 1 × cold PBS), incubated in 30% sucrose overnight, embedded in OCT, and stored at –80°C until use. Bones were sectioned in 10 μm by using the CryoStar NX50 manual cryostat (Thermo Fisher Scientific). Sections were stained with DAPI and images were acquired with Zeiss LSM880 confocal microscope.

### MSC cell isolation and culture.

Hematopoietic and nonhematopoietic cells were obtained from BM by crushing 2 femurs and 2 tibias with 2% FBS HBSS. The bone fragments were incubated with collagenase II in the 37°C water bath for 1 hour. We removed the bone fragments by using a 100 μm cell strainer, collected the digested cells, and combined them with BM cells. The cells were presorted with Lineage Depletion Kit (STEM CELL Technologies) to remove majority of differentiated cells. CD45^–^, Ter119^–^, and CD31^–^ cells were sorted by flow cytometry and cultured in α-MEM with 20% FBS (76, 77). The MSCs in vitro culture should not be over 2 months.

### Trilineage differentiation of mouse MSCs.

MSCs were seeded and allowed to reach confluency before initiating differentiation. For adipogenic and osteogenic differentiation, MSCs were cultured in StemXVivo Osteogenic/Adipogenic Base Media, while for chondrogenic differentiation, cells were cultured in StemXVivo Chondrogenic Base Media. All reagents were obtained from R&D Systems. The differentiation process was induced for 14 days for adipogenic and osteogenic differentiation and 21 days for chondrogenic differentiation. Following the differentiation period, cells were fixed with 4% formaldehyde and subjected to immunofluorescence staining using the primary antibodies: anti-FABP Antibody (R&D Systems, Cat#: AF1443-SP) for adipogenic differentiation, anti-Osteopontin (R&D Systems, Cat#: AF808-SP) for osteogenic differentiation, and anti-Collagen II (R&D Systems, Cat#: AF3615-SP) for chondrogenic differentiation. Secondary staining was performed using a Sheep IgG NorthernLights NL557-conjugated antibody (R&D Systems, Cat#: NL010). Nuclei were stained with DAPI, and an antifade reagent was added to prevent photobleaching. Images were captured using a Leica DMI8 Deconvolution Microscope.

### LSK cells coculture with MSC cells for long-term colony formation.

The sorted Lin^–^, CD45^–^, TER119^–^, CD31^–^ MSC cells (passage 0) were cultured into confluence and then seeded to the 96-well plates until they reached full confluence. The LSK cells (male or female) were sorted from preenriched lineage depleted cells and plated above the MSC cells (male or female) at doses of 6,667, 2,222, 741, 247, 82, and 28. The cobblestone colonies were observed every 7 days for 5 weeks. The cobblestone formation was assessed on day 35, representing the long-term HSC (78). The long-term colony CAFC frequencies were calculated and analyzed using L-Cac limiting dilution analysis software (STEM CELL Technologies).

### BM transplantation assay.

For the sex-mismatch competitive transplantation assay, 1 × 10^6^ donor BM cells from male or female BM (CD45.2) were mixed with an equal number of competitor BM cells of B6.SJL/BoyJ (the gender of competitor is consistent with recipient) and respectively retroorbitally injected into lethally (9Gy) irradiated male or female recipient mice (B6.SJL/BoyJ). Thus, 4 transplantation groups were set up (male to male, female to male, male to female, and female to female). Percentages of donor cells (CD45.2) in PB cells were determined at 4, 8, and 12 weeks. Percentages of donor cells in BM were determined at 12 weeks after transplantation (78).

For transplantation into CD45.1/2 chimeric mice assay, 1 × 10^6^ BM cells from the male (B6.SJL/BoyJ, CD45.1) and 1 × 10^6^ BM cells from the female (B6, CD45.2) were mixed and injected to lethally irradiated CD45.1/2 chimeric male or female mice, respectively. PB and BM were analyzed at 12 weeks after transplant.

For the assay involving BM cotransplanted with sorted mesenchymal stromal cells, 1 × 10^6^ male BM cells, 1 × 10^6^ female BM cells, 1 × 10^6^ female BM cells with 1.7 × 10^4^ male mesenchymal stromal cells (CD45^–^, CD31^–^, and Ter119^–^), and 1 × 10^6^ female BM cells with 1.7 × 10^4^ female mesenchymal stromal cells were injected with the helper cells (1 × 10^6^ BM cells from female B6.SJL/BoyJ mice) into lethally irradiated CD45.1/2 heterozygous male or female mice, respectively. PB was analyzed at 2, 5, 8, and 12 weeks. BM analysis was performed at 14 weeks after transplantation.

In the GFP^+^ MSC transplantation, GFP^+^ MSCs were isolated from the BM of male or female GFP^+^ mice and sorted to obtain 7-AAD^–^, CD45^–^, Ter119^–^, and CD31^–^ cell populations. A total of 8 × 10^5^ male or female GFP^+^ MSC cells, with 1 × 10^6^ female BM helper cells, were injected into lethally (9Gy) irradiated male B6.SJL/BoyJ mice. One week after transplantation, BM samples from the recipients were collected and analyzed for the presence of GFP^+^ cells to determine the MSC engraftment.

For the assay involving BM cotransplanted with *Kdm5c*^ΔPrrx1/X^ MSC cells, 1 × 10^6^ male BM cells, 1 × 10^6^ female BM cells, 1 × 10^6^ female BM cells with cultured 1.5 × 10^5^ female MSC cells, and 1 × 10^6^ female BM cells with cultured 1.5 × 10^5^ female *Kdm5c*^ΔPrrx1/X^ MSC cells were injected into sublethally (6.5Gy) irradiated CD45.1/2 heterozygous male or female mice, respectively. PB was analyzed at 8 weeks.

### Western blot.

The MSC cells were cultured as described above. The total proteins of MSC cells were isolated using RIPA buffer (Sigma-Aldrich) with the protease inhibitor cocktail (CST). KDM5C (Bethyl Laboratories, Cat#: A301-035A) and KDM5D (Bethyl Laboratories, Cat#: A301-751A) antibodies were bought from Bethyl Laboratories.

### ELISA.

BM supernatant was collected by flushing 2 femurs and 2 tibias with 1 mL PBS. BM cells were removed by centrifugation to obtain the clear BM plasma. To obtain the supernatant of MSC cells, the MSC cells were incubated in α-MEM with 20% FBS for 48 hours. To obtain the pure supernatant, the cell debris was removed by centrifugation. The concentration of CXCL12 was detected by using the Mouse CXCL12/SDF-1 DuoSet ELISA kit (R&D Systems) and following the manufacture protocol. The concentration of SPP1 was detected by using the Mouse Osteopontin DuoSet ELISA kit (R&D Systems) and following the manufacture protocol.

### ChIP-qPCR.

MSC cells were isolated and cultured as described above. Cells were cross-linked at a 1% final concentration of formaldehyde and incubated at room temperature for 10 minutes on a shaking platform. Crosslinking was stopped by adding 2.5M glycine at room temperature for 5 minutes. The media was removed and the plates were rinsed with cold PBS. The cells were scraped in cold PBS plus protease inhibitors and the liquid and cells were centrifuged to get the cell pellet. The pellet was resuspended with cell lysis buffer plus protease inhibitor, then incubated on ice for 10 minutes, followed by centrifugation to pellet the nuclei. The supernatant was discarded and the nuclei resuspended with Nuclei Lysis Buffer. It was then sonicated to obtain chromatin fragment lengths of 200–1,000 bp. The chromatin immunoprecipitation, antibody-protein-DNA complex recovery, washing, and reverse cross-link was accomplished using EZ-ChiP Kit (Millipore) and following the manufacture protocol. The eluted DNA was purified with DNA clean and concentrator kit (Zymo Research). ChIP-qPCR was run with the Power SYBR reagents (Thermo Fisher Scientific) on the Quant Studio Real Time PCR System. The antibodies used in ChIP-qPCR were anti-H3K4me3 (Millipore, Cat#: 07-473), and anti-KDM5C (Bethyl-Laboratories, Cat#: A301-035A). The primer sequences are as follows: mqCHIP-CXCL12-10#- forward (F), CTGCATCAGTGACGGTGAGT; mqCHIP-CXCL12-10#- reverse (R), CCTGCAGCCCTCTCTAGGT; mqCHIP-CXCL12-11#- F, GGTTTTGTGCTCTGCGAAGT; mqCHIP-CXCL12-11# -R, CCGGTCTTTGAGAGTTTGCT; mqCHIP-CXCL12-12#- F, AGATGTTTCCAGAGGCGAAG; mqCHIP-CXCL12-12# -R, GACCAACGAACTGTGCAGAA; mqCHIP-CXCL12-13#- F, GAAGTGCATGGCTTGGCTAT; mqCHIP-CXCL12-13# -R, GGGTAACTGCTGAGCCTTTG.

### The quantification of mRNA.

MSC cells were used to obtain the cDNA as described previously (79). Briefly, Total RNA was isolated by using the mirVanaTM miRNA isolation kit (Invitrogen). cDNA reverse transcription was performed by using the high-capacity cDNA reverse transcription kit (Applied Biosystems). Quantitative real-time PCR(q-RT-PCR) was performed with the commercially available Taqman probe for *Kdm5c*, *Kdm5d*, *Cxcl12*, and *Gapdh* (as the reference gene) by using the TaqMan Gene Expression Master Mix (Applied Biosystems) in ABI PRISM 7700 (Applied Biosystems).

### Knockdown of Kdm5c and Kdm5d in MSC cells by lentiviral transduction.

MSC cells were seeded in 6-well plates and incubated overnight. A total of 5–10 MOI of control or shKdm5c, shKdm5d lentivirus with polybrene was added to MSC cells. After 6 hours of incubation, the medium was changed. After another 36 hours, GFP positive cells were sorted and left to grow for a week. MSC cells or supernatant were collected for further experiments.

### Overexpression of Kdm5c using electroporation.

The overexpression-*Kdm5c* and control plasmids were synthesized by VectorBuilder. Male or female MSC were transfected using a Lonza 4D-Nucleofector system with the P3 Primary Cell 4D-Nucleofector X Kit (Lonza). Two days after transfection, RNA was extracted to assess gene expression, and culture supernatants were collected to measure the concentration of CXCL12 protein using an ELISA assay.

### In vitro cKit^+^ cell migration assay.

The migration rate of cKit^+^ cells was assessed using an in vitro transwell (5 μm pore size) assay. The cKit^+^ cells were sorted from GFP mouse BM cells using a positive anti-CD117 bead separation (Stem Cell technologies). The adherent male or female MSCs, pretreated with or without CXCL12 inhibitor AMD3100 (5 μM) for 1 hour, were cocultured for 4 hours with male or female GFP^+^/cKit^+^ cells, respectively. The migration rate was determined by calculating the ratio of the number of migrated GFP^+^/cKit^+^ cells to the total number of cells.

### scRNA-seq and analysis.

Single cells were barcoded using the 10× Chromium single-cell platform, and cDNA libraries were prepared following the manufacturer’s instructions (Chromium Single Cell 3′ Kits v3.1, 10× Genomics, USA). BM cells were harvested from 10 male C57BL/6 mice and 10 female C57BL/6 mice. Stromal cells were sorted by cell surface marker CD45^–^, Lin^–^, Ter119^–^, and 7-AAD^–^. Live cell counts were further determined using a hemocytometer. The prepared cells were subsequently loaded onto a 10x Genomics Chip, aiming for an output of approximately 10,000 cells per sample. The pooled libraries were then sequenced using the NovaSeq 6000 S4 system (Illumina), targeting 400 million reads per library. Count matrices generation was conducted using the 10x Genomics Cell Ranger pipeline (version 5.0.0), following the 10x Genomics guidelines. The demultiplexed FASTQ files were aligned to the mm 10 reference genome. Data normalization, integration, and clustering were subsequently carried out using the Seurat package (version 4.3.0) (80). Quality control filtering was applied, removing cell barcodes of low complexity. The following parameters were used for filtering: cells with less than 5% of reads mapping to the mitochondrial chromosome, Unique Molecular Identifier (UMI) counts of over 500, and a detected gene count of over 500. Defining the clusters was manually assigned and refined based on expressed genes previously reported (34, 81–83).

### Statistics.

Data were examined for homogeneity of variances (F test), then analyzed by a 2-tailed, unpaired Student’s *t* test. Statistical analyses were performed using GraphPad Prism Software version 7.0. The results shown represent mean ± SD. Differences were considered significant at *P* < 0.05.

### Study approval.

Mice were housed at the University of Kentucky animal facilities and New York Blood Center animal facilities following NIH-mandated guidelines for animal welfare and with IACUC approval. All experimental procedures followed the approved IBC protocols.

### Data availability.

The scRNA-seq dataset of BM stromal cells (CD45^–^, Lin^–^, Ter119^–^, and 7-AAD^–^) is available in the Gene Expression Omnibus database with the code GSE267695. Values for all data points in graphs are reported in the Supporting Data Values file.

## Author contributions

XC performed the majority of experiments and wrote the Methods section and figure legends for the manuscript. LH conducted all experiments for the revision, drafted the revised manuscript, and edited it to meet the journal’s publication requirements. XC and LH contributed equally to the publication of this manuscript. BY, JL, and PS were involved scRNA-seq and data analysis. CZ was involved in cell sorting experiments. LL and AM were involved in MSC characterization and HSCT. ST, DZ, FY and HZ were involved in scRNA-seq, analyses and manuscript review. YL guided the overall project, designed the experiments and wrote the manuscript.

## Supplementary Material

Supplemental data

Unedited blot and gel images

Supplemental table 1

Supplemental table 2

Supporting data values

## Version 1 01/21/2025

In-Press Preview

## Version 2 03/03/2025

Electronic publication
