# Supplementary material for: Sexual dimorphism in the mouse bone marrow niche regulates hematopoietic engraftment via sex-specific Kdm5c/Cxcl12 signaling
Source: J Clin Invest. 2025 Jan 21;135(5):e182125. doi: 10.1172/JCI182125 (PMC11870739; doi:10.1172/JCI182125)
Supplement: Supplemental data [file jci-135-182125-s098.pdf]

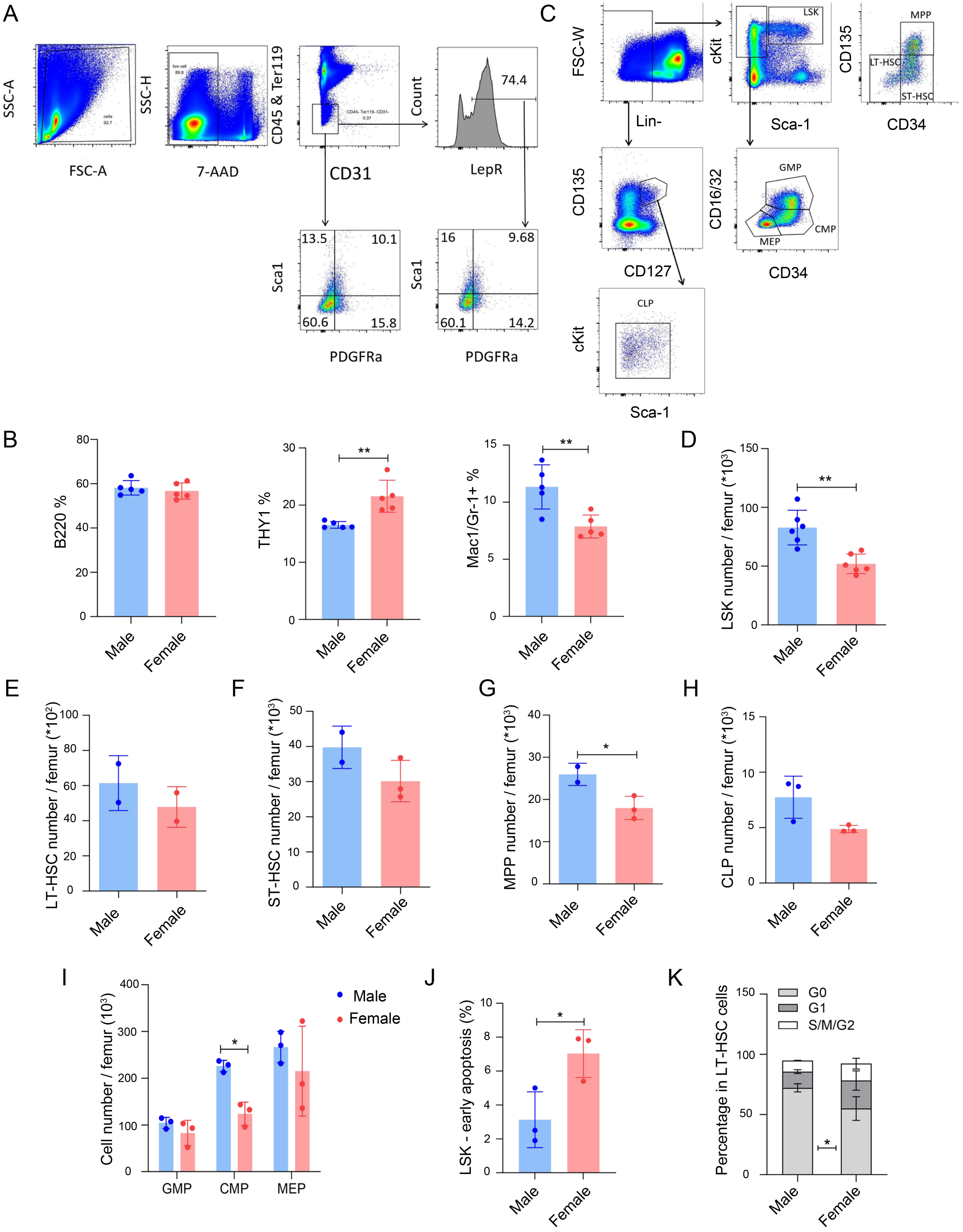

A
SSC-A
FSC-A
SSC-H
7-AAD
CD45 & Ter119
CD31
Count
LepR
74.4
Sca1
PDGFRa
13.5
10.1
60.6
15.8
16
9.68
60.1
14.2
C
FSC-W
Lin-
cKit
Sca-1
LSK
CD135
CD34
MPP
LT-HSC
ST-HSC
CD127
CD16/32
GMP
CMP
MEP
CLP
B
B220 %
THY1 %
Mac1/Gr-1+ %
Male
Female
D
LSK number / femur (*10³)
E
LT-HSC number / femur (*10²)
F
ST-HSC number / femur (*10³)
G
MPP number / femur (*10³)
H
CLP number / femur (*10³)
I
Cell number / femur (10³)
GMP
CMP
MEP
J
LSK - early apoptosis (%)
K
Percentage in LT-HSC cells
G0
G1
S/M/G2

[illegible]

Supplement Figure 1. Sexual dimorphism in the hematopoiesis.

(A) Representative fluorescence-activated cell sorting (FACS) analysis of BM stromal (LepR, Sca1, and PDGFRa) from non- hematopoietic cells (7AAD-, CD45-, Ter119- and CD31-). LepR+ or PDGFRa+/Sca1+ MSCs are separately gated from 7AAD-, CD45-, Ter119- and CD31- cells. PDGFRa+/Sca1+MSCs are also gated from LepR+ cells in order to determine their relationship. (B) FACS analysis of the percentage of different lineage cells in peripheral blood (PB) of both genders, including B lymphocytes (B220+), T lymphocytes (Thy1+) and myeloid cells (MAC1+ and Gr-1+) (male n=5; female n=5). (C) Representative FACS analysis of hematopoietic stem/progenitor cells: LSK cells are Lin-, cKit+, Sca-1+; LT-HSC cells are LSK with CD34-, CD135-; ST-HSC cells are LSK with CD34+, CD135-; MPP cells are LSK with CD34+, CD135+; CLP cells are Lin-, Sca-1^{med}, cKit^{med}, CD127+, CD135+; MEP cells are Lin-, Sca-1, cKit+, CD34-, CD16/32-; CMP cells are Lin-, Sca-1-, cKit+, CD34^{med}, CD16/32^{med}; GMP cells are Lin-, Sca-1-, cKit+, CD34+, CD16/32+. (D – H) The absolute number of (D) LSK, (E) LT-HSC, (F) ST-HSC, (G) MPP, (H) CLP cells in one femur of male (n=3-6) and female (n=3-6) mice. (I) The absolute number of CMPs, GMPs, and MEPs in one femur of male (n=3) and female (n=3) mice. (J) Percentage of apoptotic LSK cells (Annexin V+ PI-) in male (n=3) and female (n=3) mice. (K) Percentage of G0, G1, and S/M/G2 phases in the population of LT-HSCs. The data were analyzed by a two-tailed t-test and shown as mean ± SD. ** $p < 0.01$, * $p < 0.05$.

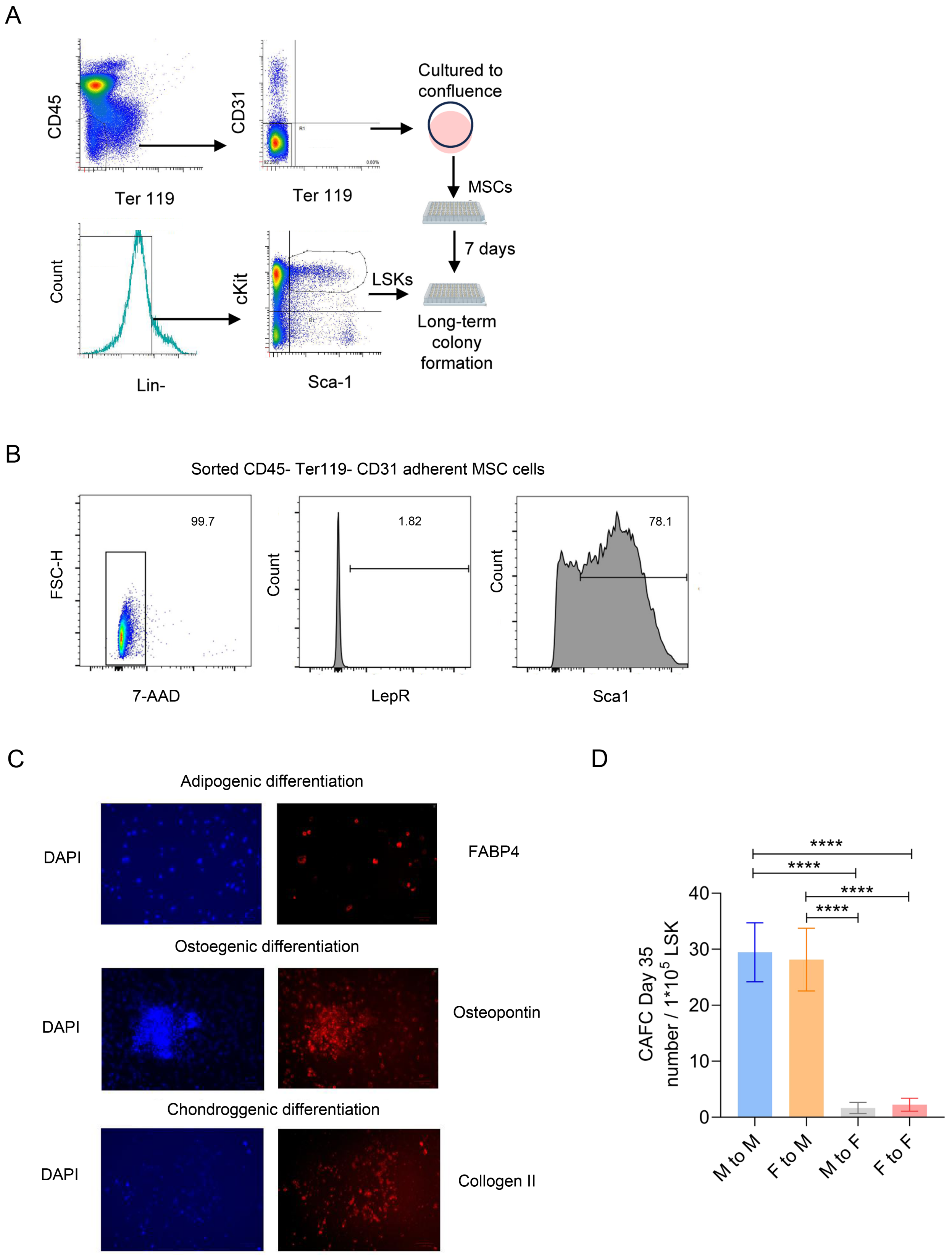
A
CD45
Ter 119
CD31
Ter 119
Cultured to confluence
MSCs
7 days
Count
Lin-
cKit
Sca-1
LSKs
Long-term colony formation
B
Sorted CD45- Ter119- CD31 adherent MSC cells
FSC-H
7-AAD
99.7
Count
LepR
1.82
Count
Sca1
78.1
C
Adipogenic differentiation
DAPI
FABP4
Ostoegenic differentiation
DAPI
Osteopontin
Chondroggenic differentiation
DAPI
Collogen II
D
CAFC Day 35 number / 1*10^5 LSK
0
10
20
30
40
****
****
****
****
M to M
F to M
M to F
F to F

Supplement Figure 2. Male BM stroma provides superior support for in vitro colony formation.

(A) Schematic illustration of the in vitro co-culture experimental workflow. (B) FACS analysis of LepR and Sca1 using sorted CD45-, Ter119-, CD31 non- hematopoietic BM cells. (C) Trilineage differentiation of adherent MSCs using immunofluorescence. Adipogenic differentiation was assessed by staining with anti-FABP4 after 14 days differentiation. Ostoegenic differentiation was assessed by staining with anti-Osteopontin after 14 days differentiation. Chondrogenic differentiation was assessed by staining with anti-Collogen II after 21 days differentiation.

(D) The absolute number of clones formed by 100k LSK cells at day 35 in co-culture with male or female MSC cells. Blue column represents male LSK seeded onto male stromal cells (M to M). Orange column represents female LSK seeded onto male stromal cells (F to M). Grey column represents male LSK seeded onto female stromal cells (M to F). Red column represents female LSK seeded onto female stromal cells (F to F).

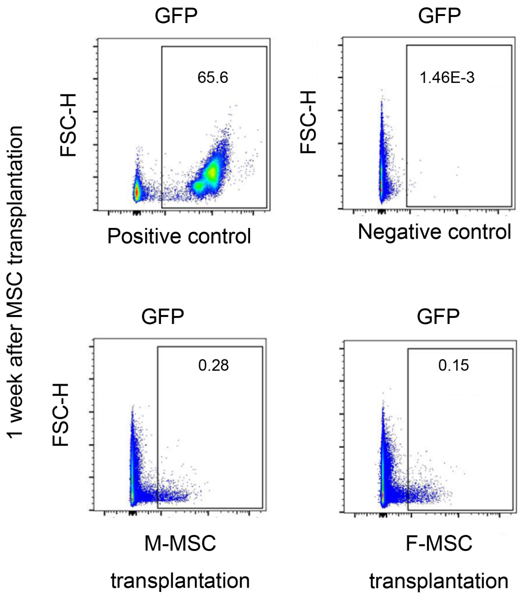
1 week after MSC transplantation
GFP
FSC-H
65.6
Positive control
GFP
FSC-H
1.46E-3
Negative control
GFP
FSC-H
0.28
M-MSC
transplantation
GFP
0.15
F-MSC
transplantation

Supplement Figure 3. More male MSC homed to the BM when co-infusion with hematopoietic cells in HSCT.

Transplanted MSC can successfully home to recipients' BM. Male or female MSCs (7AAD-, CD45-, Ter119, CD31-) from the BM of GFP+ mice were sorted using FACS and injected to the gender matched recipient mice. After 1 week of transplantation, the GFP signal in recipients' BM were detected using FACS. Top left panel is the GFP positive control. Top right panel is negative control. Bottom left shows the GFP+ BM cells (0.28%) in the male recipients transplanted with male MSCs (M-MSC). Bottom right shows the GFP+ BM cells (0.15%) in the male recipients transplanted with female MSCs (F-MSC).

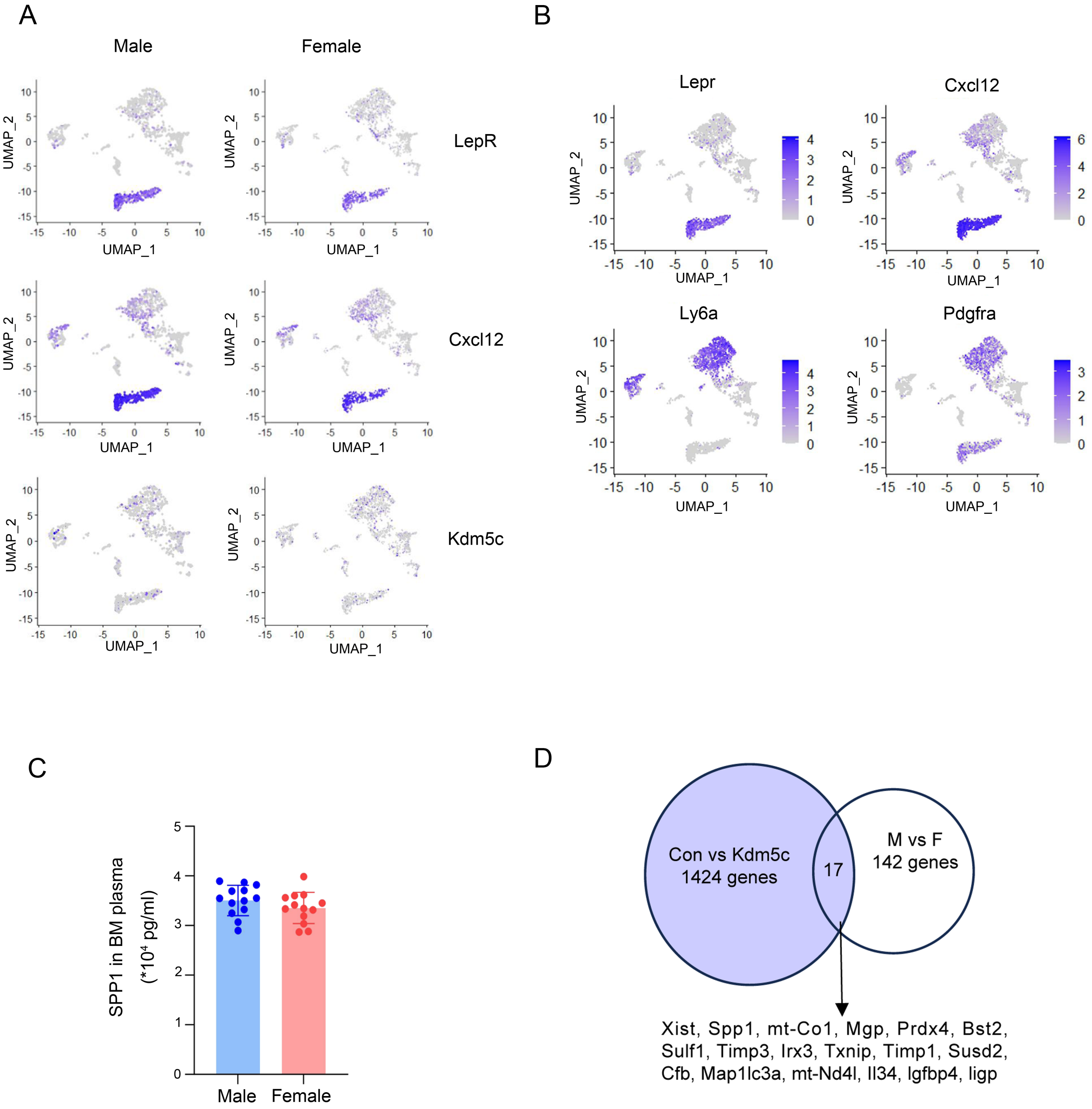
A
Male
Female
LepR
Cxcl12
Kdm5c
UMAP_1
UMAP_2
B
Lepr
Cxcl12
Ly6a
Pdgfra
UMAP_1
UMAP_2
C
SPP1 in BM plasma
(*10⁴ pg/ml)
Male
Female
D
Con vs Kdm5c
1424 genes
17
M vs F
142 genes
Xist, Spp1, mt-Co1, Mgp, Prdx4, Bst2,
Sulf1, Timp3, Irx3, Txnip, Timp1, Susd2,
Cfb, Map1lc3a, mt-Nd4l, Il34, Igfbp4, Iigp

Supplement Figure 4. Key MSC gene markers in scRNA sequencing analysis and identification of *Kdm5c* as the major regulator of sex dimorphism of BM niche.

(A) UMAP of *LepR*, *Cxcl12* and *Kdm5c* genes in both genders. (B) UMAP of key MSC marker genes (*LepR, Cxcl12, Ly6a and Pdgfra*) using merged scRNA-seq data. (C) The concentration of SPP1 in male and female BM plasma was detected by ELISA. (D) Differentially expressed genes between male and female MSCs overlapped with genes whose expression was altered by *Kdm5c* overexpression.

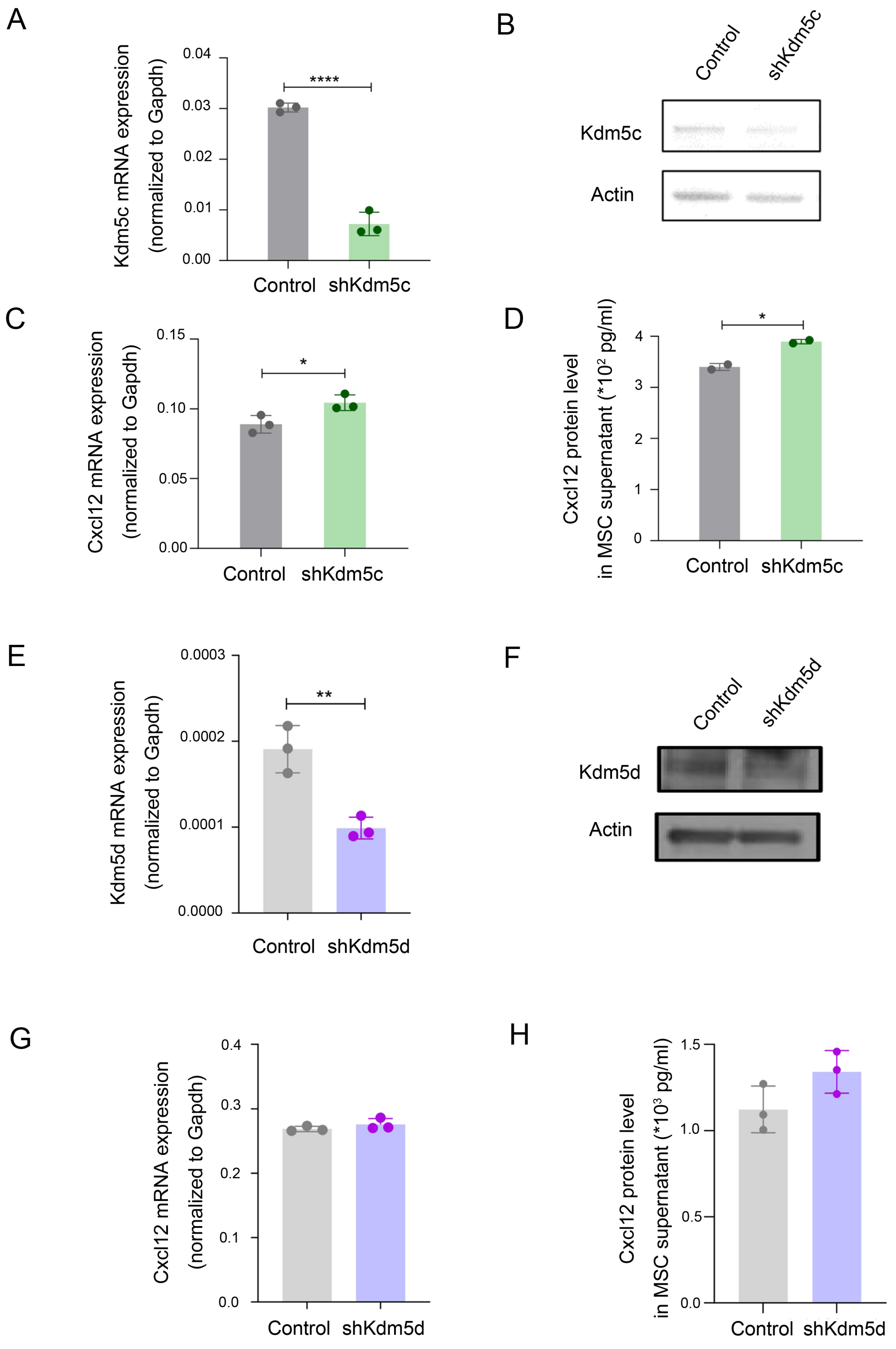
A
Kdm5c mRNA expression
(normalized to Gapdh)
0.04
0.03
0.02
0.01
0.00
****
Control
shKdm5c
B
Control
shKdm5c
Kdm5c
Actin
C
Cxcl12 mRNA expression
(normalized to Gapdh)
0.15
0.10
0.05
0.00
*
Control
shKdm5c
D
Cxcl12 protein level
in MSC supernatant (*10² pg/ml)
4
3
2
1
0
*
Control
shKdm5c
E
Kdm5d mRNA expression
(normalized to Gapdh)
0.0003
0.0002
0.0001
0.0000
**
Control
shKdm5d
F
Control
shKdm5d
Kdm5d
Actin
G
Cxcl12 mRNA expression
(normalized to Gapdh)
0.4
0.3
0.2
0.1
0.0
Control
shKdm5d
H
Cxcl12 protein level
in MSC supernatant (*10³ pg/ml)
1.5
1.0
0.5
0.0
Control
shKdm5d

Supplement Figure 5. Regulation of KDM5C and KDM5D in *Cxcl12* expression in male MSC cells.

(A) The mRNA level of *Kdm5c* in male MSC cells with control or *shKdm5c* transduction. (B) The protein level of KDM5C in male MSC cells with control or *shKdm5c* transduction. (C) The mRNA level of *Cxcl12* in male MSC cells with control or *shKdm5c* transduction. (D) The concentration of CXCL12 in the control or *shKdm5c* transduced male MSC culture supernatant was detected by ELISA. (E) The mRNA level of *Kdm5d* in male MSC cells with control or *shKdm5d* transduction. (F) The protein level of KDM5D in male MSC cells with control or *shKdm5d* transduction. (G) The mRNA level of *Cxcl12* in male MSC cells with control or *shKdm5d* transduction. (H) The concentration of CXCL12 in control or *shKdm5d* transduced male and female MSC culture supernatant was detected by ELISA. All the data were derived from 2 independent experiments with 2-3 replicates from each experiment, are shown as mean ± SD, and analyzed by two-tailed t-test. *** is $p < 0.001$, ** $p < 0.01$, * $p < 0.05$.

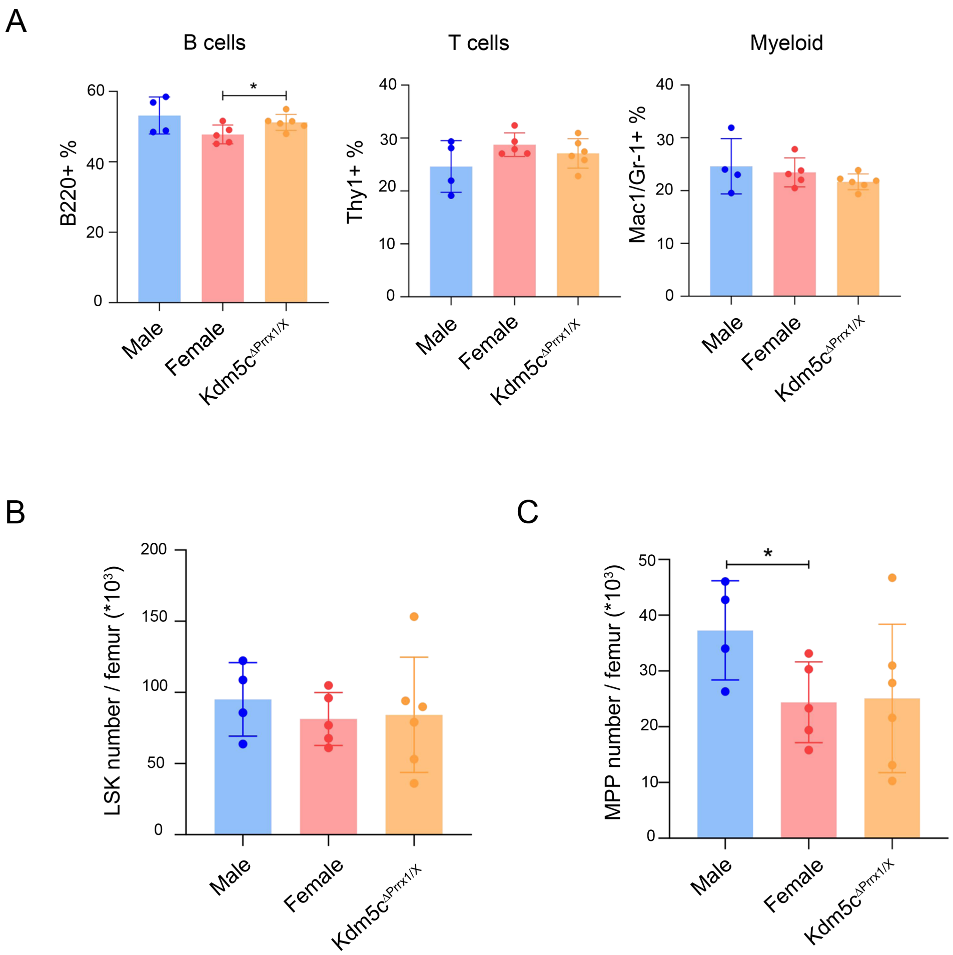
A
B cells
T cells
Myeloid
B220+ %
Thy1+ %
Mac1/Gr-1+ %
0
20
40
60
0
10
20
30
40
*
Male
Female
Kdm5cΔPrrx1/X
B
C
LSK number / femur (*10³)
MPP number / femur (*10³)
0
50
100
150
200
0
10
20
30
40
50

Supplement Figure 6. Female *Kdm5c*$^{\Delta Prrx1/X}$ mice show no significant changes in the blood cells and BM HSPC populations.

(A) Lineage differentiation analysis by staining cell surface markers and conducting flow cytometry in male (*Kdm5c*$^{loxp/Y}$), female (*Kdm5c*$^{loxp/X}$), and female *Kdm5c*$^{\Delta Prrx1/X}$ mice: B lymphocytes, B220+; T lymphocytes, Thy1+; and Myeloid cells, MAC1+ and Gr-1+ (n=4-6). (B-C) The absolute number of (B) LSK, (C) MPP in one femur of male (*Kdm5c*$^{loxp/Y}$, n=4), female (*Kdm5c*$^{loxp/X}$, n=5), and female *Kdm5c*$^{\Delta Prrx1/X}$ mice (n=6). The data were analyzed by a two-tailed t-test and shown as mean ± SD. * $p < 0.05$.

Supplement Table 1. Genes expressed in the BM stromal cells and genes differentially expressed between male and female stromal cells and their GO enrichment analysis.

There are 370 different expressed genes (bold font) were between male and female stromal cells based on the adjust P value. Over one thousand pathways were involved in regulating the gender differences in bone marrow niche via GO enrichment analysis. Data Attached in Excel.

Supplementary Table 2. Differentially expressed genes out of all detected genes in each cellular components of male and female stromal cells and their GO enrichment analysis.

Different expressed genes (DEG) and GO enrichment pathways were analyzed between male and female in MSC, Chondrocytes, BMEC, Adipocyte, Osteo-lineage cells, Fibroblast, and Megakaryocyte, and differentially expressed genes between male and female stromal cells are highlighted with bold font. Data Attached as Excel.
