# Supplementary figures and images for: Sexual dimorphism in the mouse bone marrow niche regulates hematopoietic engraftment via sex-specific Kdm5c/Cxcl12 signaling
Source: J Clin Invest. 2025 Jan 21;135(5):e182125. doi: 10.1172/JCI182125 (PMC11870739; doi:10.1172/JCI182125)

Fig 5B

Actin

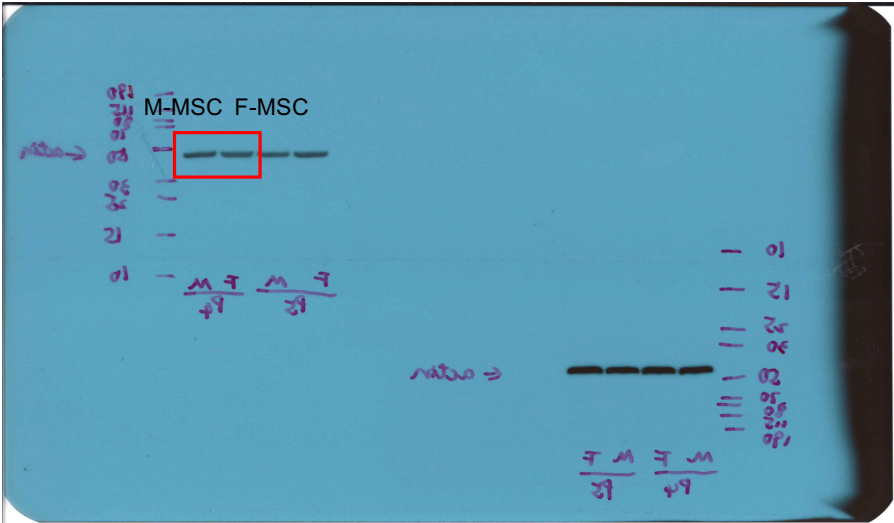

Kdm5c

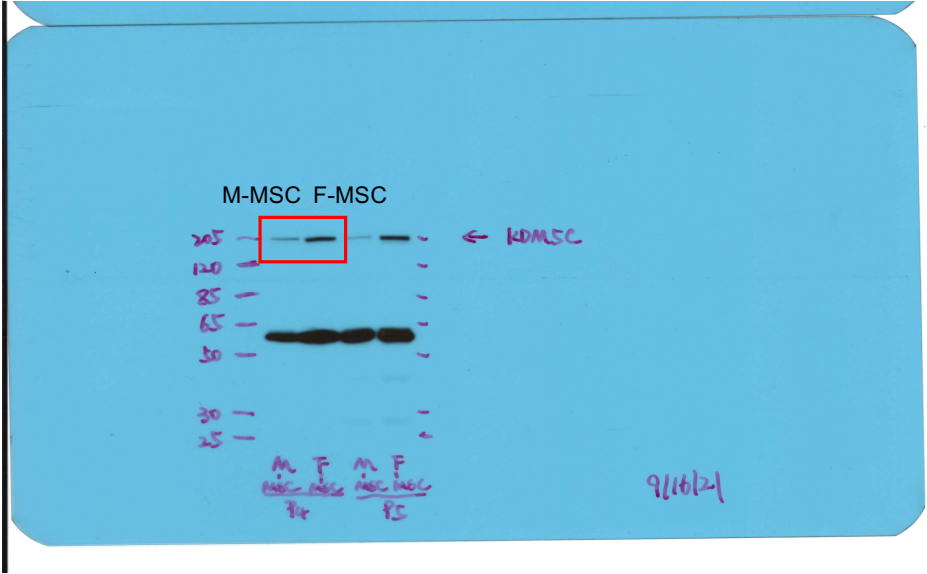

Fig 5F

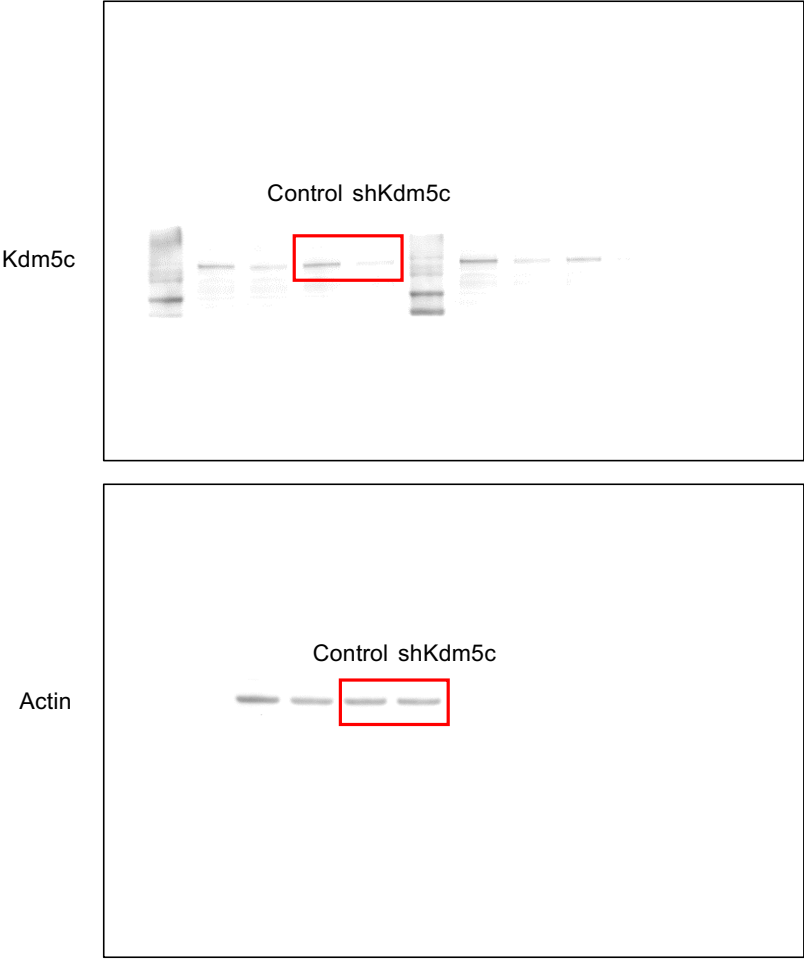

Fig 6B

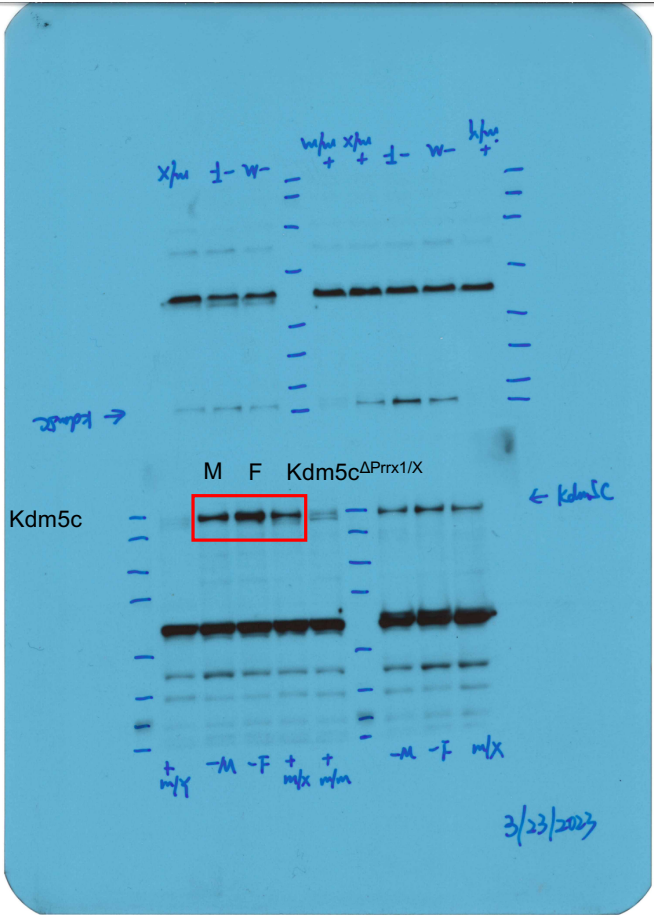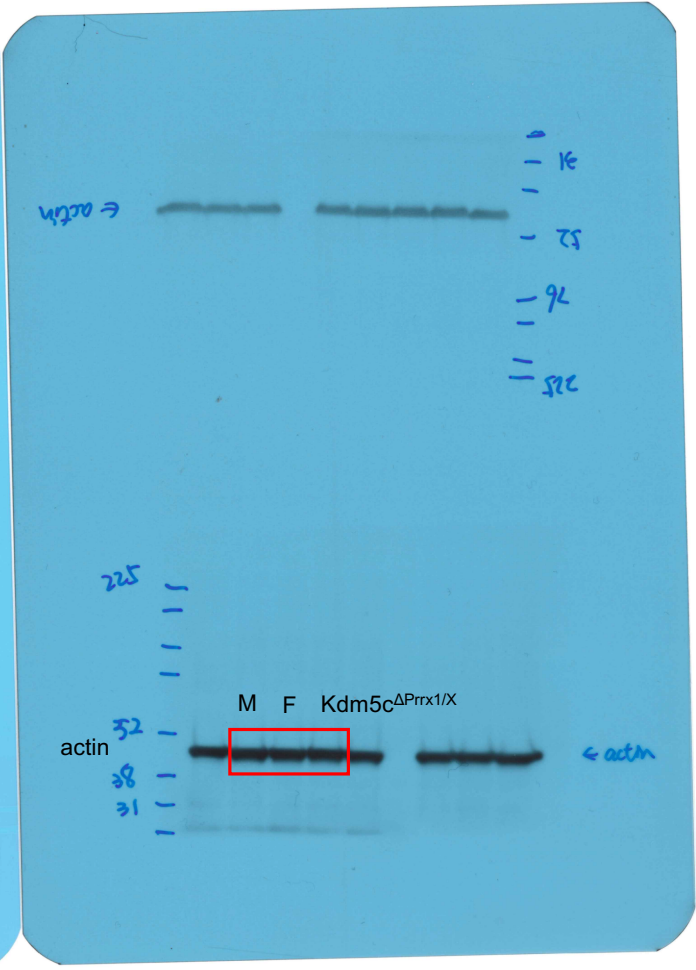

Sup Fig 4B

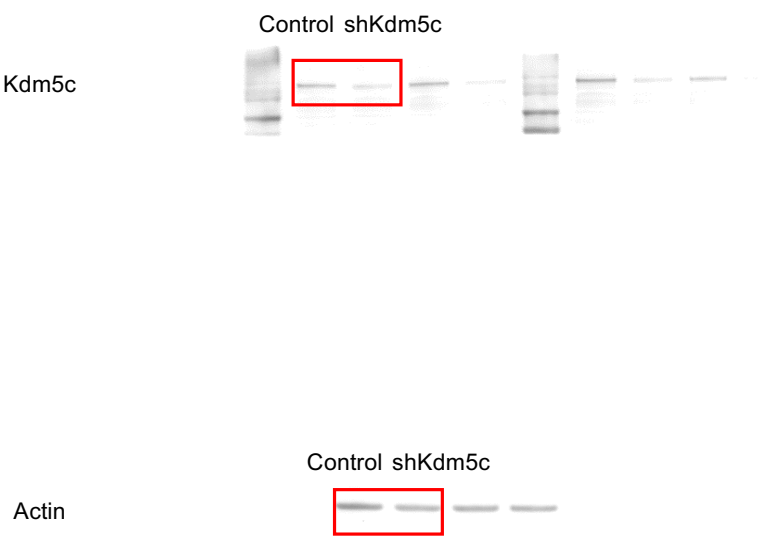

Sup Fig 4F

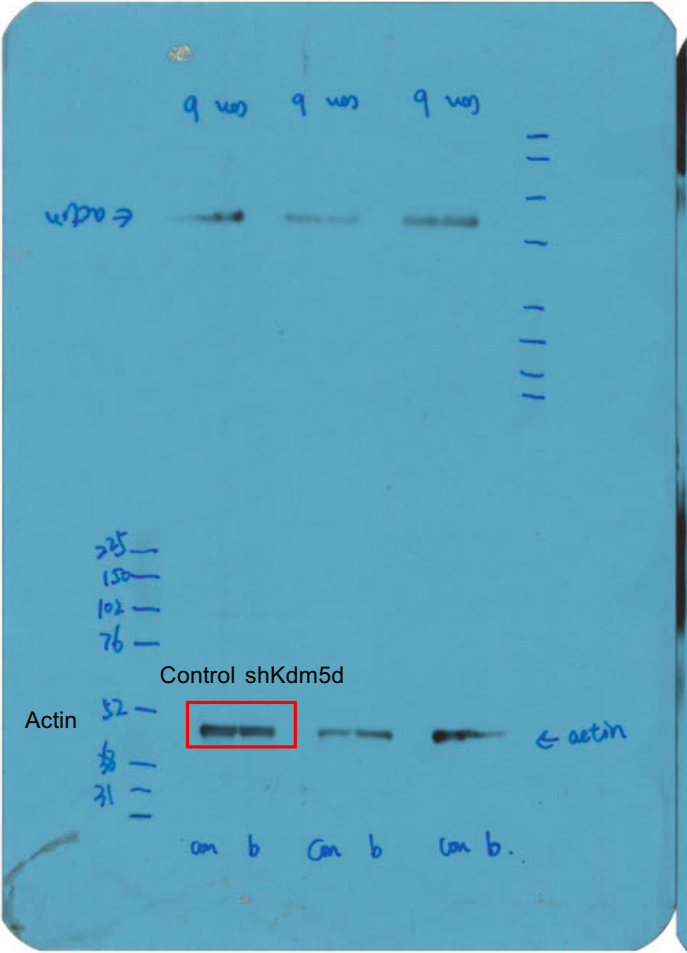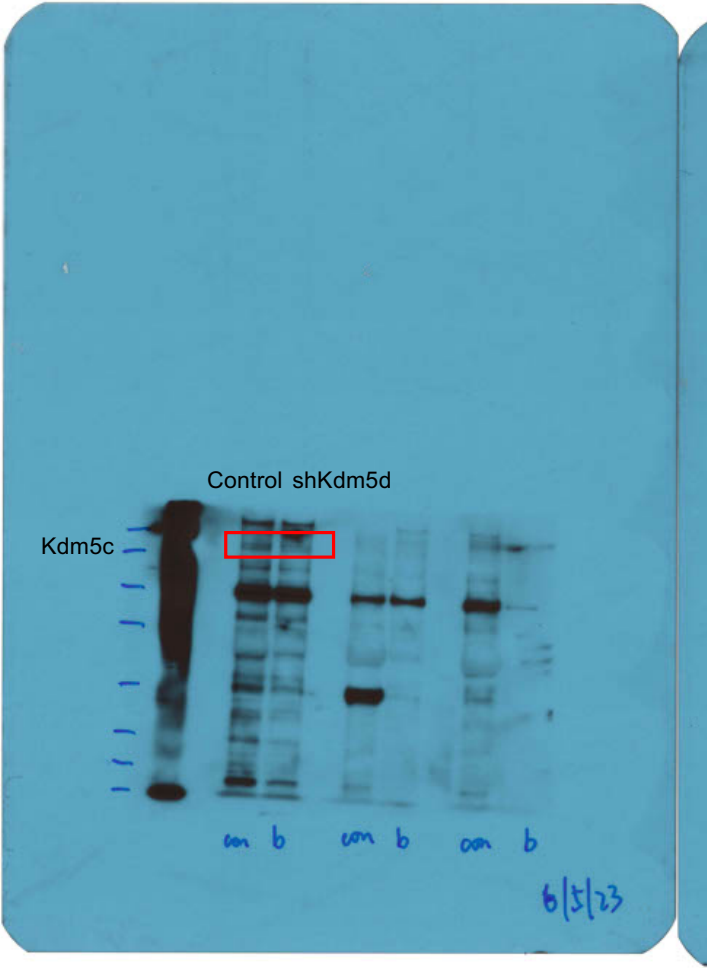

Supplement: Unedited blot and gel images [file jci-135-182125-s099.pdf]
